# Metabolomics profiling of AKT/c-Met-induced hepatocellular carcinogenesis and the inhibitory effect of Cucurbitacin B in mice

**DOI:** 10.3389/fphar.2022.1009767

**Published:** 2022-11-23

**Authors:** Xiangyu Ji, Xin Chen, Lei Sheng, Dongjie Deng, Qi Wang, Yan Meng, Zhenpeng Qiu, Baohui Zhang, Guohua Zheng, Junjie Hu

**Affiliations:** ^1^ College of Pharmacy, Hubei University of Chinese Medicine, Wuhan, Hubei, China; ^2^ Key Laboratory of Chinese Medicine Resource and Compound Prescription, Ministry of Education, Hubei University of Chinese Medicine, Wuhan, Hubei, China

**Keywords:** hepatocellular carcinoma, AKT/c-Met, Cucurbitacin B, metabolomics, UPLC-Q-TOF-MS/MS

## Abstract

Hepatocellular carcinoma (HCC), the most common kind of liver cancer, accounts for the majority of liver cancer diagnoses and fatalities. Clinical aggressiveness, resistance to traditional therapy, and a high mortality rate are all features of this disease. Our previous studies have shown that co-activation of AKT and c-Met induces HCC development, which is the malignant biological feature of human HCC. Cucurbitacin B (CuB), a naturally occurring tetracyclic triterpenoid compound with potential antitumor activity. However, the metabolic mechanism of AKT/c-Met-induced Hepatocellular Carcinogenesis and CuB in HCC remains unclear. In this study, we established an HCC mouse model by hydrodynamically transfecting active AKT and c-Met proto-oncogenes. Based on the results of hematoxylin-eosin (H&E), oil red O (ORO) staining, and immunohistochemistry (IHC), HCC progression was divided into two stages: the early stage of HCC (3 weeks after AKT/c-Met injection) and the formative stage of HCC (6 weeks after AKT/c-Met injection), and the therapeutic effect of CuB was evaluated. Through UPLC-Q-TOF-MS/MS metabolomics, a total of 26 distinct metabolites were found in the early stage of HCC for serum samples, while in the formative stage of HCC, 36 distinct metabolites were found in serum samples, and 13 different metabolites were detected in liver samples. 33 metabolites in serum samples and 11 in live samples were affected by CuB administration. Additionally, metabolic pathways and western blotting analysis revealed that CuB influences lipid metabolism, amino acid metabolism, and glucose metabolism by altering the AKT/mTORC1 signaling pathway, hence decreasing tumor progression. This study provides a metabolic basis for the early diagnosis, therapy, and prognosis of HCC and the clinical application of CuB in HCC.

## Introduction

Approximately 75%–85% of all occurrences of primary liver cancer are caused by HCC, which is also the sixth most frequent cancer worldwide and the third major cause of cancer-related death (Duran and Jaquiss, 2019; Craig et al., 2020). HCC has an onset that is gradual and stealthy, with high metastasis, high malignancy, and a poor prognosis, all of which pose a major threat to the health of humans (Aravalli et al., 2008; Pinzani et al., 2011). Sorafenib, Lenvatinib, and regorafenib are among the most commonly used drugs for the treatment of HCC (Bruix et al., 2017) with high-cost and low-survival rate. Alpha-fetoprotein (AFP) is a commonly used early detection marker for HCC with limited clinical value (Ryder and Gastroenterology, 2003; Parikh and Hyman, 2007). Therefore, it is of great significance to search for HCC biomarkers and effective drugs with high specificity and sensitivity.

Complex and diverse molecular regulatory mechanisms are closely related to metabolite dynamics. Previous studies have shown that mutation or abnormal expression of proto-oncogenes causes deregulation of cell signaling pathways, which mediates abnormal cell proliferation or inhibits programmed cell death and eventually forms HCC, which is one of the main mechanisms of hepatocellular carcinogenesis. HCC is frequently associated with aberrant activation of the proto-oncogenes AKT and c-Met as well as their overexpression. AKT/c-Met causes rapid HCC development by activating the AKT/mTORC1 signaling pathways (Hu et al., 2016). The co-expression of p-AKT and c-Met has a high incidence in the occurrence of HCC, which is the malignant biological feature of human HCC. HCC with abnormal activation of AKT/mTORC1 and c-Met could be effectively suppressed by inhibiting p-AKT and c-Met. Nevertheless, only the molecular mechanism by which AKT/c-Met facilitates the development of HCC in mice has been identified, leaving the metabolic level unknown.

**FIGURE 1:**
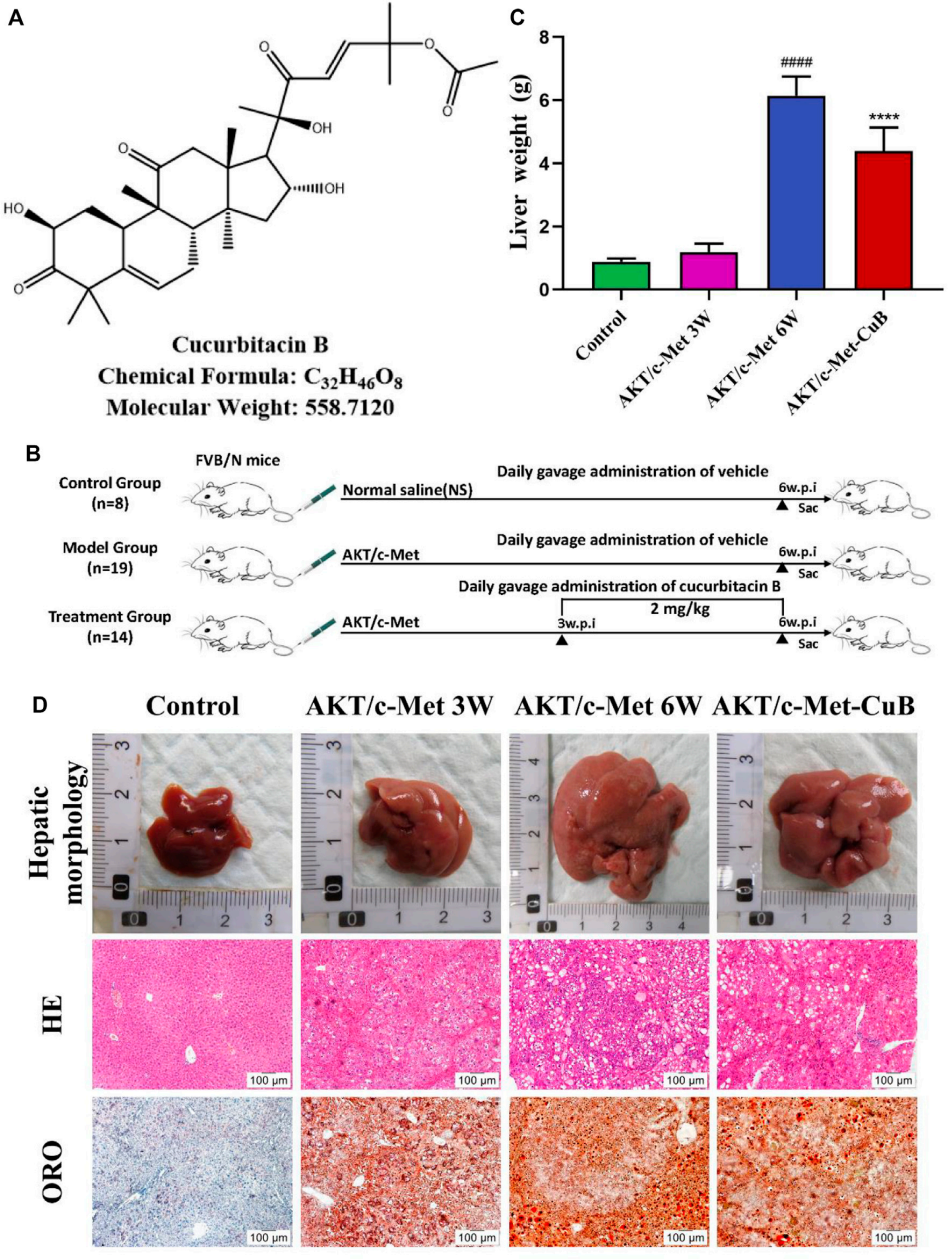
Co-expression AKT/c-Met-triggered rapid hepatocarcinogenesis and the inhibitory effect of CuB in mice. **(A)** CuB structure, chemical formula, and molecular weight; **(B)** Design of research process; **(C)** Liver weight of mice in each group (Mean ± S.D., *n* = 3. ^####^ *p* < 0.0001 vs. Control group; ****p < 0.0001 vs. AKT/c-Met group, pounds and asterisks indicate statistical significance as determined by two-tailed unpaired *t*-test comparisons with two groups); **(D)** Hepatic morphology, HE, and ORO results of mice in each group (Magnification: ×100. w.p.i., weeks post-injection).

Recent years, searching for small molecule anticancer substances from medicinal plants has become a possible way to prevent and inhibit liver cancer (Teufel et al., 2009). CuB (Figure 1A) is a class of tetracyclic triterpenoids from *Lagenaria siceraria var. depressa,* which belongs to *Cucurbitaceae* and possesses anti-inflammatory, antiviral, hepatoprotective, and immunomodulatory properties (Lee et al., 2010; Gupta and Srivastava, 2014). CuB also has significant antitumor effects on a variety of malignant tumors, and its mechanism of action involves multiple tumor phenotypes and signaling pathways (Kalimuthu et al., 2020). Previous research has demonstrated that CuB can enhance plasma cAMP levels, modify the concentration of hemoproteins, increase the cAMP/cGMP ratio, ameliorate the aberrant metabolism of alanine aminotransferase (ALT) caused by liver damage, and stimulate the regeneration of injured hepatocytes (Yang and Zheng, 2006). In addition, CuB induces apoptosis and inhibits the growth of BEL-7402/5-Fu HCC cells by preventing their S and G0/G1 stages (Chan et al., 2010; Sun et al., 2015). *In vitro* studies have shown that CuB inhibits hepatocellular cancer, although there has been no investigation into the metabolic levels of this compound.

Metabolomics has been utilized extensively to investigate the endogenous metabolites of an organism associated with the early identification of diseases and to obtain a deeper understanding of disease pathophysiology and treatment targets by examining these biomarkers (Wang S. et al., 2022). Understanding the metabolic network of drug targets resulting from inhibitions in a holistic context provides a new way to screen for reliable biomarkers (Lu et al., 2021). UPLC-Q-TOF-MS/MS combines the advantages of high separation capacity of ultra-high performance liquid chromatography and high sensitivity and specificity of mass spectrometry, and is considered to be the most stable and universal platform for metabolomics because of its relatively simple sample processing method and large analyte coverage. Using metabolomics approaches, this work aims to evaluate the mechanisms of AKT/c-Met-induced hepatocellular carcinogenesis and the inhibitory effect of CuB in mice. To provide a potentially valuable metabolic basis for the early diagnosis, treatment and prognosis of clinical HCC patients and the clinical application of CuB in HCC.

## Materials and methods

### Drugs and reagents

CuB (purity > 98.0%, CAS: 6199-67-3) was procured from Pusi Biotechnology Co., Ltd. (Chengdu, China). Sinopharm supplied ethyl alcohol, xylene, chloroform, and isopropanol (Beijing, China). Merck supplied acetonitrile (CAS: 75-05-8) and methanol (CAS: 57-55-1) of MS grade (Darmstadt, Germany). Mreda supplied formic acid of HPLC grade (CAS: 64-18-6) (Beijing, China). Shanghai Yuanye Biotechnology Co., Ltd. supplied 2-chloro-L-phenylalanine (HPLC ≥ 98%, CAS: 103616-89-3) (Shanghai, China). Abcam provided phosphorylated mammalian target of rapamycin (p-mTOR) and β-actin antibodies (Cambridge, United States). Cell Signaling Technology supplied the following antibodies: total protein kinase B (t-AKT), phosphorylated protein kinase B (p-AKT^Thr308^), phosphorylated ribosomal protein S6 (p-RPS6), fatty acid synthase (FASN), acetyl-CoA carboxylase (ACC), hexokinase 2 (HK2), and pyruvate kinase M2 (PKM2) (Danvers, United States). ABclonal supplied the antibody to sterol regulatory element-binding protein (SREBP1) (Wuhan, China). Details are shown in Supplementary Table S1. Dr. Xin Chen of the University of California, San Francisco, California, United States, donated pT3-EF1-HA-myr-AKT, pT3-EF1-V5-c-Met, and pCMV/sleeping beauty transposase (pCMV-SB). Plasmids were isolated and purified using an E.Z.N.A.^®^ Endo-Free Plasmid Maxi Kit prior to being injected into mice (Omega Bio-Tek, Norcross, GA, United States).

### Hydrodynamic transfection and Cucurbitacin B treatment

Si Pei Fu (Beijing, China) provided wild-type (WT) FVB/N mice, permit number SCXK (Kyoto) 2019-0010. All animal studies were carried out on mice aged 6–7 weeks. Water and mouse chow were available to the mice at all times, and mice were housed in a facility with a 12-h light/dark cycle. By combining hydrodynamic injection with sleeping beauty-mediated somatic integration, Dr. Xin Chen devised a method for generating stable gene expression in the mouse liver that is useful for the generation of murine HCC models (Hu et al., 2016). After 1 week of habitat adaptation, 41 mice were randomly assigned to either the control group (8) or the AKT/c-Met-induced HCC group (33). To evaluate the therapeutic efficacy, AKT/c-Met-induced HCC mice (14) were administered 2 mg/kg CuB by gavage every day for 3 weeks following plasmid injection (Figure 1B). Both the control and model groups received the same amount of solvent. All animal experiments were carried out in accordance with the policy approved by the animal ethics committee of Hubei University of Chinese Medicine.

### Sample preparation

A safe, simple, and reproducible submandibular venous blood collection method was used for this experiment. Half of the mice in the model group were randomly selected, and 0.1–0.3 ml of blood was obtained from the submandibular vein after the mice were fasted for more than 12 h after 3 weeks of AKT/c-Met-induced plasmid injection. A half-hour period at 4°C followed by 10 min of centrifugation at 4,000 r/min was used to store and process the blood. Aliquots of the supernatant were stored in a freezer at −80°C for future use. The removed livers were weighed, and the proteins in some liver tissues were denatured and coagulated by storage in tissue fixative at 4°C. The remaining liver tissue was preserved at −80°C for later use. The tumor growth of each group of mice was observed regularly. When an abdominal bulge, hard liver, or depression could be felt, the mice were killed, and blood was taken from the submandibular vein before they were put down. After the last gavage, all mice were killed, and blood and liver tissue were collected and processed as described above.

### Hematoxylin-eosin and oil red O staining

For histological evaluation, liver tissues were fixed in 4% paraformaldehyde, embedded in paraffin, and sectioned at 5 μm thickness. The sections were then dried and mounted on coverslips for microscopic analysis.

Frozen liver tissue slices were stained with a 0.5% w/v ORO solution in propylene glycol, rinsed in 60% isopropanol and counterstained with hematoxylin to reveal the location of the nuclei on the cell surface. The slides were then placed on coverslips using aqueous mounting media and photographed under a microscope.

### Metabolomic analysis

Twenty microliters of each sample was added to 60 μl of ice-cold methanol: acetonitrile (v/v = 1:1) containing 0.5 μg/ml 2-chloro-L-phenylalanine (IS), vortexed and cultivated at 4°C for 10 min and then centrifuged at 12,000 r/min at 4°C for 15 min. The supernatant was placed in the injection vial to be analyzed. In addition, equal volumes of all thawed serum samples were mixed and processed in the same way as the quality control (QC) samples.

The lyophilized liver tissue (100 mg) of each group was added to 300 μl of ice-cold methanol, fully homogenized until there were no fibrous particles, and incubated for half an hour at 4°C. The mixture was centrifuged at 12,000 r/min and 4°C for 15 min after incubation. To dry the supernatant, nitrogen was used to blow out any remaining moisture. The residue was sonicated at 4°C for 15 min with 100 μl of methanol containing an internal standard. Centrifugation was performed to remove the supernatant. A QC sample was created by combining 10 μl of each sample. The QC sample was introduced every 10 needles during the injection procedure to ensure that the LC-MS apparatus was stable and reproducible.

Serum and liver samples were separated chromatographically using UPLC equipment (1290, Agilent Technologies, United States). At 40°C and 0.3 ml/min, serum and liver were injected into an Infinity Lab Poroshell 120 EC-C18 column (3.0 mm × 150 mm, 2.7 μm). Water with 0.1% formic acid (A) and acetonitrile (B) were used as the mobile phases. The following gradient conditions were used for the serum sample: 5%–10% B from 0 to 5 min; 10%–30% B from 5 to 9 min; 30%–60% B from 9 to 11 min; 60%–75% B from 11 to 25 min; 75%–98% B from 25 to 27 min. The liver sample’s gradient conditions were as follows: 5%–10% B from 0 to 5 min; 10%–35% B from 5 to 10 min; 35%–90% B from 10 to 30 min; 90%–98% B from 30 to 32 min.

An Agilent 6540 Q-TOF (Agilent Technologies, United States) with both positive and negative ion modes was used to acquire mass data. The sheath gas was 280°C, and the drying gas was 320°C. Other conditions followed the best analysis conditions of our research team (Chen et al., 2022).

Profinder (Agilent’s specialized software) was used to perform preprocessing on the raw serum and liver metabolomics data collected by UPLC-Q-TOF-MS/MS. To perform multivariate statistical analysis, we used Simca-P14.0 (Umetrics) software, which included principal component analysis (PCA), partial least squares discriminant analysis (PLS-DA), and orthogonal partial least squares discriminant analysis (OPLS-DA). Significance analysis was performed for compounds with at least one group retained in 80% of the samples, and compounds with significant differences (VIP > 1) were screened. Concurrently, the preprocessed data were converted into files that contained certain templates, and then those files were entered into Mass Profiler Professional software. T tests and fold tests were conducted in accordance with a specific technique, and both were combined to confirm substances with statistically significant differences [corrected *p*-value < 0.05, fold change (FC) value ≥ 1.2].

Finally, in combination with secondary fragment ions, the potential biomarkers were structurally identified using the METALIN database, the Human Metabolomics Database, etc. The MetaboAnalyst 5.0 online website (http://www.metaboanalyst.ca/), literature review, and the KEGG database were used to search for metabolic pathways and biochemical processes.

### Western blotting and immunohistochemistry

Western blotting analysis was carried out in accordance with the procedures outlined in the previous section (Mo et al., 2020). SDS-PAGE and polyvinylidene fluoride membranes were used to separate the protein lysates. The membranes were blocked for 1 h in Tris-buffered saline containing 0.1% Tween 20 before being probed with specific antibodies. Primary antibodies included p-AKT^Thr308^, t-AKT, p-mTOR, p-RPS6, SREBP1, FASN, ACC, HK2, and PKM2. Anti-rabbit/mouse IgG/HRP (secondary antibody) was added following incubation with each primary antibody. HRP-conjugated β-actin antibody from Proteintech (Wuhan, China) was used as an internal standard. Using SuperSignal West Pico Chemiluminescent Substrate (Pierce Chemical, Ridgewood, New York, United States) and a chemiluminescence imager, immunoblotting was performed and photographed (G: BOX Chemi XRQ; Syngene, Cambridge, United Kingdom).

The IHC assay was performed as previously described (Hu et al., 2016). Specific primary antibodies used in immunohistochemical staining are listed in Supplementary Table S1.

### Statistical analysis

The data were analyzed with Prism 7.0 software (GraphPad Software Inc., San Diego, CA, United States). The data from at least three different experiments were used to define the mean ± standard deviation (SD). Two-tailed unpaired *t*-test was used to compare two groups. It was established that *p* < 0.05 was a statistically significant value.

## Results

### Biomarkers discovery of AKT/c-Met-induced hepatocellular carcinoma model

**FIGURE 2:**
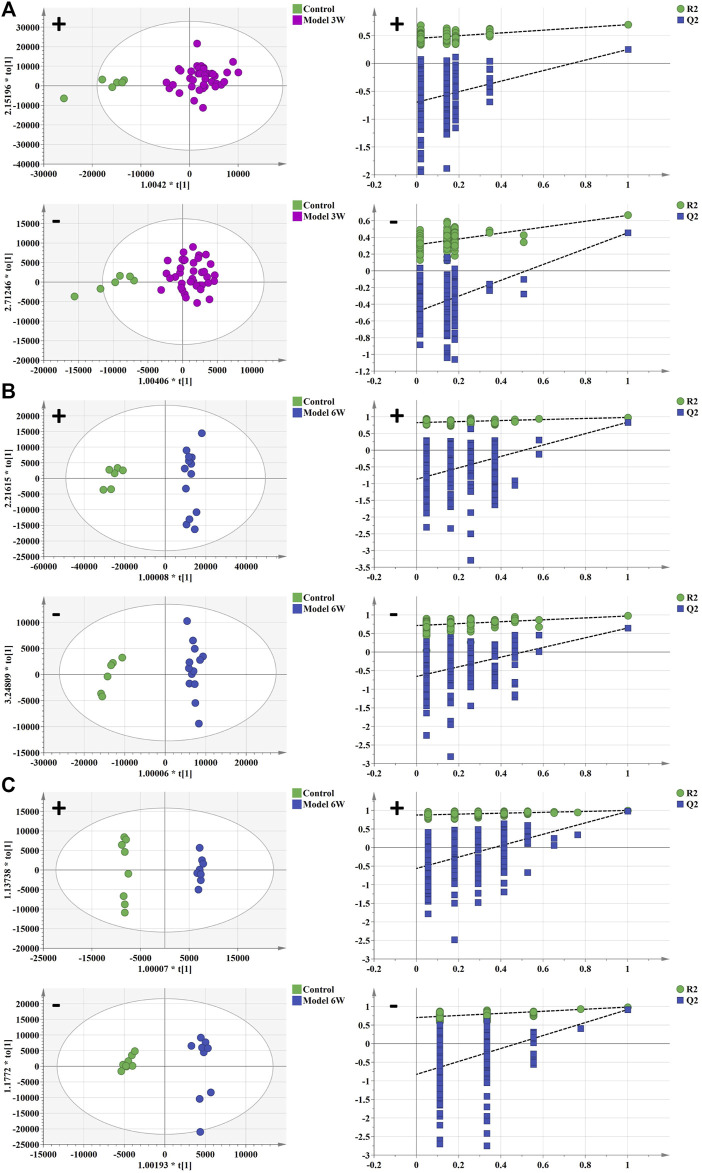
The OPLS-DA analysis and permutation test of serum and liver in positive and negative ion mode of control group, early stage of HCC, and formative stage of HCC. **(A,B)** serum metabolism profile and **(C)** liver metabolism profile. Green spot represents the control group, purple spot represents the early stage of HCC, and blue spot represents the formative stage of HCC.

Weight changes and morphological results of livers in different groups are displayed in Figures 1C,D. The AKT/c-Met group’s liver weight increased considerably relative to the control group as the weeks progressed. 3 weeks after hydrodynamic transfection, livers of AKT/c-Met mice displayed numerous hepatic steatosis, but no tumors were detected. At 6 weeks, a large hepatic tumor burden was observed in liver, covering about 70% of the total area. The results of histomorphology, H&E and ORO staining are consistent with previous results (Hu et al., 2016). According to the morphology and histopathology of the liver whether the tumor appears, AKT/c-Met-induced HCC could be divided into two stages: the early stage of HCC (3 weeks after AKT/c-Met injection) and the formative stage of HCC (6 weeks after AKT/c-Met injection). We then tested the proliferation profile changes with an IHC experiment. The IHC assay suggested that AKT/c-Met promoted the expression of proliferation markers Ki67 and PCNA, as shown in Supplementary Figure S1. TIC profiles in serum and liver were analyzed using positive and negative ion modes for control samples and early and formative stages of HCC, as shown in Supplementary Figures S2–S4. The results for repeatability and stability of the equipment and the analysis method are shown in Supplementary Tables S2, S3. As demonstrated in Supplementary Figure S5, PCA and PLS-DA analyses of serum and liver were performed in positive and negative ion modes to differentiate the metabolic features of various groups. The clustering of QC samples in the PLS-DA plots indicated that the analytical system was stable. Moreover, the metabolic profiles of the groups at different stages were separated from each other. The tendency of the model group to move away from the control group was more obvious as the number of weeks increased, suggesting that the serum metabolic profiles of AKT/c-Met mice after hydrodynamic transfection were disturbed. The OPLS-DA and the permutation test for the positive and negative ion models among the control group, and each stage of HCC are shown in Figure 2. The statistical model established for the permutation test had an R2 intercept less than 0.3 and a Q2 less than 0.05, and the values of Q2 and R2 were lower than the original points, indicating that the established partial least squares model was successful, with good accuracy and predictability, as shown in Supplementary Table S4.

To further explore the variability between different groups, compounds with VIP > 1, *p* < 0.05, and FC ≥ 1.2 were considered potential HCC differential metabolites. Consequently, UPLC-Q-TOF MS/MS analysis found a total of 55 divergent metabolites, 42 in serum samples and 13 in liver samples, and classified them as possible biomarkers linked with AKT/c-Met-induced HCC. In the early stage of HCC, the abundance levels of 26 metabolites were significantly altered in serum samples relative to the control group, whereas the abundance levels of 36 metabolites were significantly altered in the formative stage of HCC. The results of the identification are shown in Table 1. The parent ions and associated fragment ions of the metabolites are depicted in Supplementary Figure S6. 20 shared metabolites were screened from the differential metabolites at each developmental stage of HCC in serum samples, which were found throughout the development of HCC with significant changes in metabolite levels, reflecting the pathophysiological changes at different stages of HCC formation, and were considered biomarkers of hepatocellular carcinogenesis. In the formative stage of HCC, the abundance levels of 13 metabolites were significantly altered in liver samples compared to the control group. The results of the identification are shown in Table 2. The parent ions and associated fragment ions of metabolites are depicted in Supplementary Figure S7.

**TABLE 1:** Representative differential metabolites in serum of control, model and CuB group.

No.	RT	Metabolite	Formula	MW	HMDB ID	ESI mode	MS/MS fragments	Change trend		
								a	b	c
1	2.24	Succinylacetoacetate	C_8_H_10_O_6_	202.0456	HMDB0240258	+	[203.0528]	-	↓	-
2	2.26	Bergapten	C_12_H_8_O_4_	216.0404	HMDB0030637	−	[215.0332] [141.0374]	-	↓	-
3	2.51	Aminopropylcadaverine	C_8_H_21_N_3_	159.1247	HMDB0012189	+	[160.1811] [101.1104]	-	↑	↓
4	2.93	6,8-Dihydroxypurine	C_5_H_4_N_4_O_2_	152.0330	HMDB0001182	+	[153.0408] [110.0350] [108.0205]	-	↑	↓
5	3.18	Hypoxanthine	C_5_H_4_N_4_O	136.0386	HMDB0000157	+	[137.0458] [119.0343] [110.0349]	↑	↑	↓
6	3.20	Inosine	C_10_H_12_N_4_O_5_	268.0807	HMDB0000195	−	[267.0739] [135.0315] [108.0203]	↑	-	↓
7	3.45	L-Norieucine	C_6_H_13_NO_2_	131.0946	HMDB0001645	+	[132.1016] [104.0778]	-	↑	↓
8	8.55	Indoleacrylic acid	C_11_H_9_NO_2_	187.0637	HMDB0000734	+	[188.0707] [170.0590] [128.0490]	↑	-	↓
9	12.75	Taurallocholic acid	C_26_H_45_NO_7_S	515.2917	HMDB0000922	−	[514.2857] [496.2763]	-	↑	↓
10	14.31	Deoxycytidine	C_9_H_13_N_3_O_4_	227.1883	HMDB0000014	+	[228.0948] [132.0528]	-	↓	↑
11	16.44	Succinic acid	C_4_H_6_O_4_	118.0782	HMDB0000254	+	[119.0333]	-	↑	↓
12	16.47	4-O-Methylmelleolide	C_24_H_30_O_6_	414.2044	HMDB0037039	+	[415.2115] [397.2046]	↓	-	↓
13	16.47	N-Eicosapentaenoyl glutamic acid	C_25_H_37_NO_5_	431.2312	HMDB0242070	+	[175.1084] [130.0581] [103.0546]	↓	-	↓
14	17.37	LysoPC(16:1(9Z)/0:0)	C_24_H_48_NO_7_P	493.3165	HMDB0010383	+	[494.3241] [476.3111] [104.1068]	↑	-	↑
15	18.14	LysoPE(22:6(4Z,7Z,10Z,13Z,16Z,19Z)/0:0)	C_27_H_44_NO_7_P	525.2856	HMDB0011526	+	[526.2935] [385.2725] [311.2344]	↑	↑	↓
16	18.25	LysoPE(24:6(6Z,9Z,12Z,15Z,18Z,21Z)/0:0)	C_29_H_48_NO_7_P	613.3387	HMDB0011529	−	[552.3093] [152.9970]	↑	↑	↓
17	18.34	LysoPC(22:6(4Z,7Z,10Z,13Z,16Z,19Z)/0:0)	C_30_H_50_NO_7_P	567.3324	HMDB0010404	+	[568.3389] [184.0725] [104.1072]	↑	↑	↓
18	18.34	Guanosine diphosphate mannose	C_16_H_25_N_5_O_16_P_2_	605.2825	HMDB0001163	+	[241.0074] [121.0509]	↑	↑	↓
19	18.35	LysoPE(20:4(8Z,11Z,14Z,17Z)/0:0)	C_25_H_44_NO_7_P	501.2856	HMDB0011518	+	[502.2931] [361.2740] [119.0850]	↑	↑	↓
20	18.44	PE(22:4(7Z,10Z,13Z,16Z)/0:0)	C_27_H_48_NO_7_P	589.3383	LMGP02050014	−	[528.3098] [152.9951]	↑	↑	↓
21	18.54	LysoPC(20:4(8Z,11Z,14Z,17Z)/0:0)	C_28_H_50_NO_7_P	543.3326	HMDB0010396	+	[544.3399] [526.3279] [184.0735]	↑	↑	↓
22	18.57	LysoPC(0:0/18:2(9Z,12Z))	C_26_H_50_NO_7_P	519.3324	HMDB0061700	+	[520.3398] [502.3268] [184.0732]	-	↑	↓
23	18.58	Acrimarine N	C_32_H_31_NO_8_	557.2836	HMDB0040791	+	[380.1447]	-	↑	-
24	18.58	LysoPC(20:5(5Z,8Z,11Z,14Z,17Z)/0:0)	C_28_H_48_NO_7_P	541.3040	HMDB0010397	+	[542.3219] [184.0733] [104.1072]	↑	↑	↓
25	19.36	LysoPC(15:0/0:0)	C_23_H_48_NO_7_P	481.3166	HMDB0010381	−	[480.3099] [224.0690] [152.9960]	↑	-	↑
26	19.37	PS(20:2(11Z,14Z)/0:0)	C_26_H_48_NO_9_P	609.3249	LMGP03050021	−	[548.2997]	↑	-	-
27	19.45	LysoPE(22:2(13Z,16Z)/0:0)	C_27_H_52_NO_7_P	533.2825	HMDB0011522	+	[534.3581] [109.1007]	↑	↑	↓
28	19.46	LysoPC(0:0/16:0)	C_24_H_50_NO_7_P	495.3326	HMDB0240262	+	[496.3399] [478.3259] [184.0734]	↑	↑	↓
29	20.09	LysoPE(16:0/0:0)	C_21_H_44_NO_7_P	453.2850	HMDB0011503	+	[454.2930] [436.2812] [313.2738]	↑	↑	↓
30	20.19	LysoPC(20:3(8Z,11Z,14Z)/0:0)	C_28_H_52_NO_7_P	545.3487	HMDB0010394	+	[546.3546] [528.3390] [184.0725]	↑	↑	↓
31	20.77	LysoPC(22:5(7Z,10Z,13Z,16Z,19Z)/0:0)	C_30_H_52_NO_7_P	569.3480	HMDB0010403	+	[570.3543] [184.0731] [104.1068]	-	↑	↓
32	21.08	Elaidic carnitine	C_25_H_47_NO_4_	425.3500	HMDB0006464	+	[426.3573] [265.2531] [247.2397]	↑	↑	↓
33	21.53	PS(21:0/0:0)	C_27_H_54_NO_9_P	567.3540	LMGP03050026	−	[566.3461] [171.0076] [152.9948]	-	↑	↓
34	21.55	PE(14:0/14:0)	C_33_H_66_NO_8_P	635.3408	HMDB0008821	−	[152.9933]	-	↑	-
35	21.68	LysoPC(18:1(9Z)/0:0)	C_26_H_52_NO_7_P	521.3484	HMDB0002815	+	[522.3555] [184.0730] [104.1068]	↑	↑	↓
36	21.68	Adenosine diphosphate ribose	C_15_H_23_N_5_O_14_P_2_	559.2988	HMDB0001178	+	[103.0382]	↑	↑	↓
37	23.19	LysoPC(20:2(11Z,14Z)/0:0)	C_28_H_54_NO_7_P	547.3634	HMDB0010392	+	[548.3710] [365.3045] [104.1071]	-	↑	-
38	23.24	LysoPC(17:0/0:0)	C_26_H_54_NO_7_P	509.3477	HMDB0012108	+	[510.3549] [124.9982] [104.1064]	-	↑	-
39	26.00	LysoPE(0:0/18:0)	C_23_H_48_NO_7_P	481.3159	HMDB0011129	+	[482.3243] [341.3037] [216.0609]	-	↑	↓
40	26.36	Protoporphyrin IX	C_34_H_34_N_4_O_4_	561.3148	HMDB0000241	+	[563.2646] [503.2495]	↑	↑	↓
41	26.36	Platelet-activating factor	C_26_H_54_NO_7_P	523.3640	HMDB0062195	+	[524.3701] [341.3097] [184.0727]	↑	↑	↓
42	27.73	PC(18:1(9Z)e/2:0)	C_28_H_56_NO_7_P	549.3792	HMDB0011148	+	[550.3864] [367.3217] [184.0726]	↑	↑	-

RT, retention time; a, control group vs. early stage of HCC; b, control group vs. formative stage of HCC; c, formative stage of HCC vs. CuB group; “↑” mean up; “↓” mean down; “-” mean not detected.

**TABLE 2:** Representative differential metabolites in liver of control, model and CuB group.

No.	RT	Metabolite	Formula	MW	HMDB ID	ESI mode	MS/MS fragments	Change trend	
								a	b
1	2.49	Pipecolic acid	C_6_H_11_NO_2_	129.0787	HMDB0000070	+	[130.0863] [102.0555]	↑	↑
2	3.02	L-Methionine	C_5_H_11_NO_2_S	149.0507	HMDB0000696	+	[150.0585] [133.0316] [102.0550]	↓	↑
3	3.18	3-Hydroxybutyrylcarnitine	C_11_H_21_NO_5_	247.1419	HMDB0013127	+	[248.1486] [189.0754]	↑	-
4	3.21	Phenylpyruvic acid	C_9_H_8_O_3_	164.0464	HMDB0000205	+	[165.0541] [119.0486] [103.0540]	↓	↑
5	3.22	L-Tyrosine	C_9_H_11_NO_3_	181.0739	HMDB0000158	+	[182.0811] [147.0435] [123.0445]	↓	↑
6	3.33	Hypoxanthine	C_5_H_4_N_4_O	136.0383	HMDB0000157	+	[137.0458] [119.0338] [110.0344]	↓	↑
7	5.11	S-Acetyldihydrolipoamide-E	C_10_H_19_NO_2_S_2_	249.0859	HMDB0006878	+	[250.0933] [232.0805] [190.0699]	↓	↑
8	5.77	Pantetheine 4′-phosphate	C_11_H_23_N_2_O_7_PS	358.0955	HMDB0001416	−	[357.0891] [226.0488] [181.0276]	↓	↑
9	8.50	Glutamylarginine	C_11_H_21_N_5_O_5_	303.1678	HMDB0028813	+	[304.1747] [245.1005] [227.0898]	↓	↑
10	22.41	Sphingosine	C_18_H_37_NO_2_	299.2822	HMDB0000252	+	[300.2895] [282.2785] [264.2673]	↑	↓
11	23.66	LysoPE(18:2(9Z,12Z)/0:0)	C_23_H_44_NO_7_P	477.2846	HMDB0011507	+	[478.2925] [337.2745] [263.2325]	↓	-
12	23.76	LysoPE(20:4(8Z,11Z,14Z,17Z)/0:0)	C_25_H_44_NO_7_P	501.2852	HMDB0011518	+	[502.2931] [363.2836] [361.2732]	↓	↓
13	27.98	N-Nervonoyl Serine	C_27_H_51_NO_4_	453.3811	HMDB0242099	+	[454.3890] [123.1165] [109.1003	↑	↓

RT, retention time; a, control group vs. formative stage of HCC; b, formative stage of HCC vs. CuB group; “↑” mean up; “↓” mean down; “-” mean not detected.

### Metabolic pathways of AKT/c-Met-induced hepatocellular carcinoma model

**FIGURE 3:**
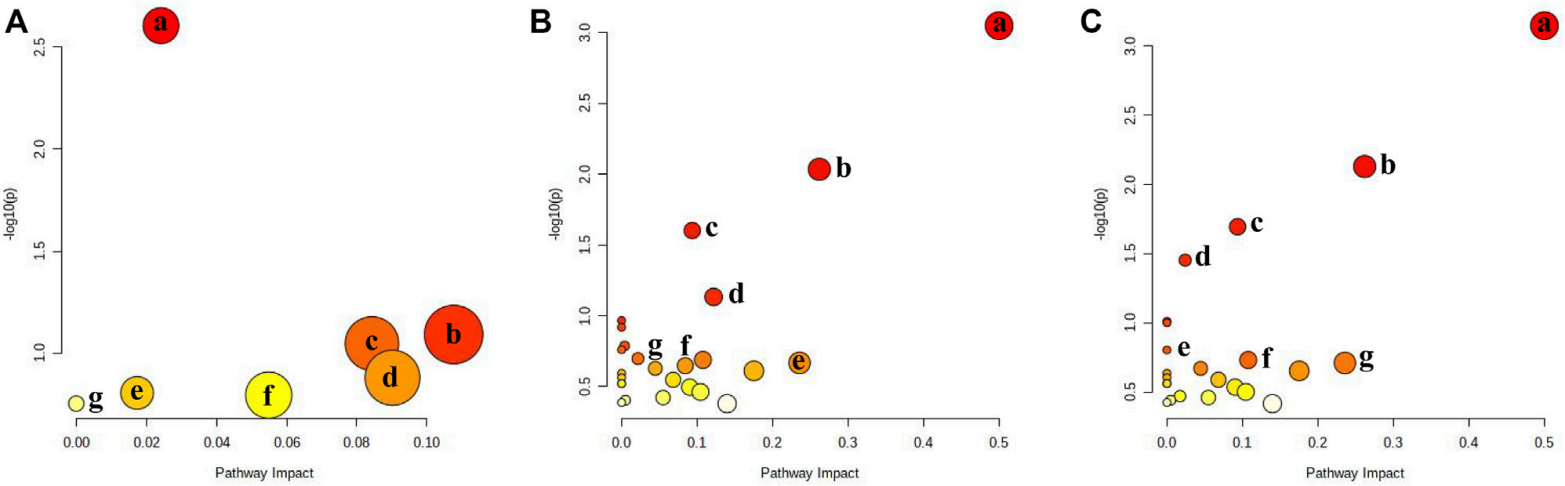
Metabolic pathway analysis for Control group vs. different stages. **(A)** Control group vs. early stage of HCC: a: Purine metabolism; b: Fructose and mannose metabolism; c: Ether lipid metabolism; d: Porphyrin and chlorophyll metabolism; e: Glycerophospholipid metabolism; f: Amino sugar and nucleotide sugar metabolism; g: N-Glycan biosynthesis; **(B)** Control group vs. formative stage of HCC: a: Phenylalanine, tyrosine, and tryptophan biosynthesis; b: Phenylalanine metabolism; c: Citrate cycle; d: Glycerophospholipid metabolism; e: Pantothenate and CoA biosynthesis; f: Ether lipid metabolism; g: Sphingolipid metabolism; **(C)** formative stage of HCC vs. CuB group: a: Phenylalanine, tyrosine, and tryptophan biosynthesis; b: Phenylalanine metabolism; c: Citrate cycle; d: Purine metabolism; e: Butanoate metabolism; f: Fructose and mannose metabolism; g: Pantothenate and CoA biosynthesis.

To clarify the process of HCC development, metabolomics pathway analysis (MetPA) was used to analyze the most significant metabolic disorders based on prospective biomarkers. Figure 3 shows the findings of an analysis of pathways performed with MetaboAnalyst 5.0 on a variety of distinct groups. KEGG enrichment analysis showed three major metabolic pathways (*p* < 0.05), primarily purine metabolism, phenylalanine metabolism, and phenylalanine, tyrosine, and tryptophan biosynthesis (Supplementary Figure S8). They were selected as key metabolic pathways associated with HCC metabolic disturbance. The heatmap analysis of serum and liver samples is shown in Supplementary Figure S9.

During the early stages of HCC, 7 metabolic pathways were dramatically changed compared to those in healthy controls (Figure 3A). The most prevalent metabolites were involved in purine metabolism, glycerophospholipid metabolism, fructose and mannose metabolism, ether lipid metabolism, porphyrin and chlorophyll metabolism, amino sugar and nucleotide sugar metabolism, and N-glycan biosynthesis. Purines are essential components of cell proliferation. Disturbances in purine metabolism may increase the burden of mutagenic diamino bases in DNA and interfere with gene expression and RNA function and are therefore closely associated with cancer progression. Glycerophospholipid metabolism is highly associated with cell membranes and signaling pathways. Lysophosphatidylcholine (LysoPC) contributes to the creation of DNA, RNA, and proteins, hence playing a key role in glycerophospholipid metabolism. LysoPC(20:2(11Z,14Z)/0:0), LysoPC(20:3(8Z,11Z,14Z)/0:0) and other LysoPC metabolites were significantly upregulated in glycerophospholipid metabolism, and this abnormal metabolic function is considered to be a metabolic feature associated with cancer progression. Linoleic acid is a precursor of arachidonic acid and is closely associated with inflammation. Lysophosphatidylethanolamine (LysoPE) is an important phospholipid on cell membranes. The level of LysoPE (16:0/0:0), a metabolite of linoleic acid, was significantly upregulated, indicating that the uptake and metabolism of linoleic acid are disrupted, which leads to abnormal body immune function and promotes tumor formation in mice through various physiological regulation processes. These findings suggest that purine metabolism, lipid metabolism, and inflammation have major roles in the pathogenic alterations that occur in mice during the early stage of HCC.

During the formative stage of HCC, 23 metabolic pathways differed significantly from the control group (Figure 3B). The major metabolites were involved in the metabolism of glycerophospholipids, sphingolipids, biosynthesis of phenylalanine, tyrosine, and tryptophan, phenylalanine metabolism, lysine degradation, and alanine, aspartate, and glutamate metabolism. According to the findings, sphingosine levels are significantly elevated, and sphingolipid metabolism is altered, which may have an impact on the proliferation and migration of cancer cells and the function of the immune system, therefore contributing to liver cancer growth (Miura et al., 2021). Furthermore, phosphatidylserine (PS) is also a classical cell signaling pathway transduction molecule, especially in the process of apoptosis. When compared to the control group, PS (21:0/0:0) levels in the model group were much higher than those in the control group, and glycerophospholipid metabolism was significantly upregulated, which may be linked to tumor formation. The tyrosine metabolic pathway is a crucial part of biological processes that is involved in making neurotransmitters, hormones, and melanin. Previous studies have shown that the end products of this metabolic pathway are involved in lipid biosynthesis, implying crosstalk between metabolite-mediated tyrosine and lipid metabolism (Wang J. et al., 2022). The results suggest that the pathogenic changes occurring in the formative stage of HCC in mice predominantly included lipid metabolism, amino acid metabolism, and glucose metabolism. To produce biomolecules (lipids, proteins, and nucleotides), it is usually assumed that signaling pathways for glucose and amino acids are integrated through the so-called mTOR pathway (Ben-Sahra and Manning, 2017; Condon and Sabatini, 2019).

### Efficacy evaluation of Cucurbitacin B

Compared with the AKT/c-Met group, the CuB group showed significantly lower liver weight (Figure 1C), significantly smaller liver volume (Figure 1D). The area covered by the tumor was reduced. H&E and ORO staining results (Figure 1D) showed a significant improvement in lipid accumulation accompanied by slight inflammatory cell infiltration and the appearance of some preneoplastic lesions, which tended to approach the early stage of HCC. The IHC assay suggested that CuB treatment profoundly repressed the proliferative potential of hepatocytes, as indicated by Ki67 and PCNA staining, with a significant decrease in number of positive cells from AKT/c-Met group (Supplementary Figure S1). This research shows that CuB has the potential to suppress the development of HCC.

### Effect of Cucurbitacin B on biomarkers

**FIGURE 4:**
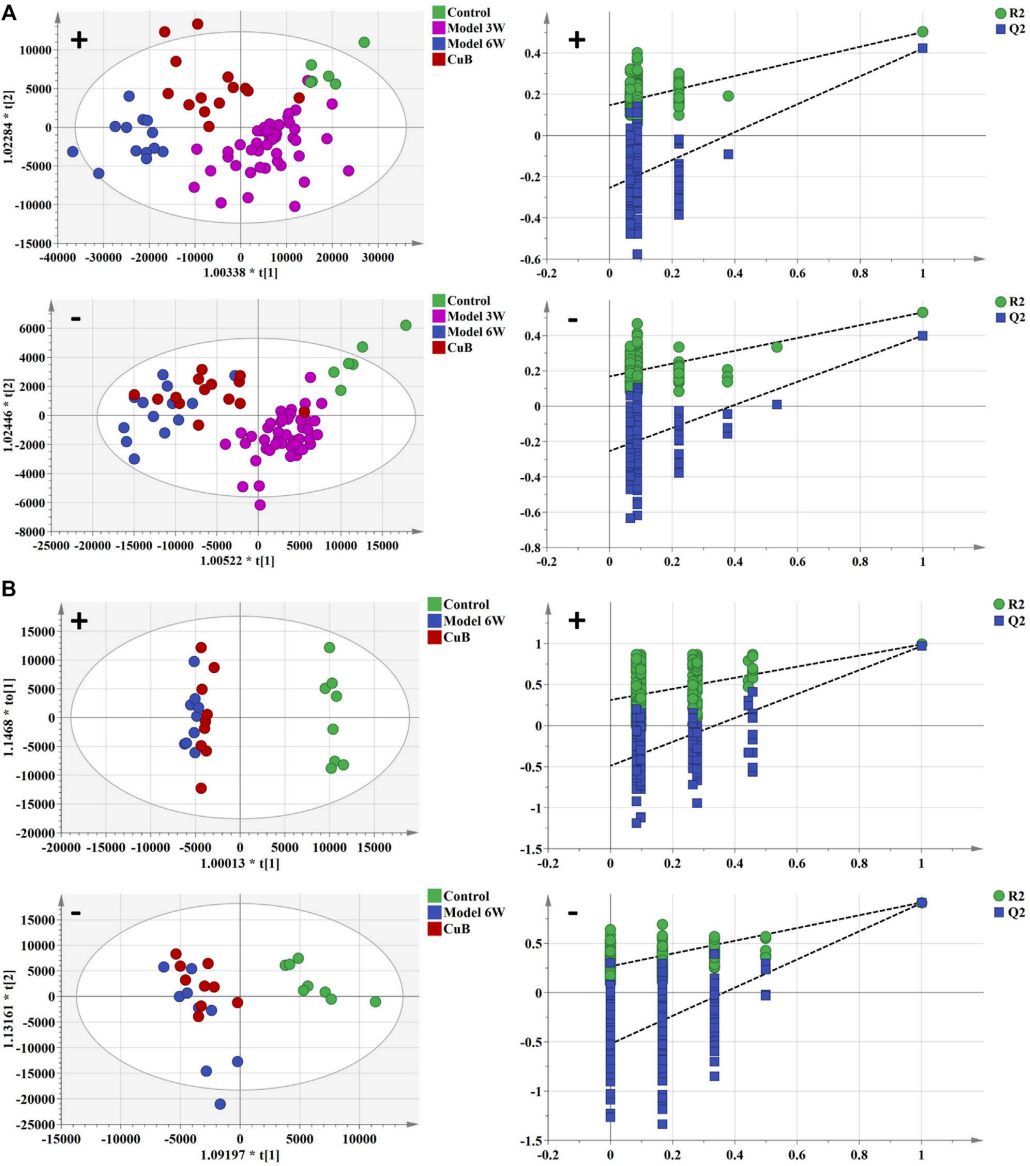
The OPLS-DA analysis and permutation test of serum and liver in positive and negative ion mode of control group, early stage of HCC, formative stage of HCC, and CuB group. **(A)** serum metabolism profile and **(B)** liver metabolism profile. Green spot represents the control group, purple spot represents the early stage of HCC, blue spot represents the formative stage of HCC and red spot represents the CuB group.

TIC profiles in serum and liver were analyzed using positive and negative ion modes for CuB treatment, as shown in Supplementary Figure S10. As demonstrated in Supplementary Figure S5, the CuB group’s similarity to the control group suggests that CuB exerted a possible therapeutic effect on HCC-related metabolic abnormalities. The OPLS-DA analysis and the permutation test for the positive and negative ion models in the CuB group are shown in Figure 4.

After CuB administration, among the 55 potential biomarkers that were characterized, the abundance levels of 33 metabolites in serum samples and 11 in liver samples changed significantly, with some significantly back-regulated compared to the model group. The results of the identification are shown in Tables 1, 2.

### Metabolic pathways of Cucurbitacin B treatment

KEGG enrichment analysis showed two major metabolic pathways (*p* < 0.05), primarily phenylalanine metabolism and phenylalanine, tyrosine, and tryptophan biosynthesis (Supplementary Figure S8). They were selected as key metabolic pathways associated with CuB back-regulation. The heatmap analysis of serum and liver samples is shown in Supplementary Figure S11.

23 metabolic pathways were significantly altered during CuB administration compared to the formative stage of HCC (Figure 3C), back-regulating most of the metabolic pathways affected during the formative stage of HCC. Previous research has demonstrated that unusually increased taurocholic acid levels are directly linked to the development of HCC (Xie et al., 2016). High levels of taurocholic acid can induce upregulation of the expression of cancer-related inflammatory genes. Long-term exposure to excessive taurocholic acid may lead to persistent DNA damage, apoptosis, and inflammation in hepatocytes, thereby increasing the risk of HCC induction. The decrease in taurocholic acid content after the action of CuB may be related to the downregulation of bile acid metabolism. Glycolysis, an important pathway of cellular energy metabolism, is often abnormal in tumor cells. Since energy is required for rapid cell proliferation, tumor cells break down glucose to lactate through glycolysis even under aerobic conditions. S-Acetyldihydrolipoamide-E, the level of which was significantly back-regulated after CuB administration, may inhibit the rapid proliferation of HCC cells by regulating glycolysis, thereby suppressing the development of HCC.

### Metabolic network analysis

**FIGURE 5:**
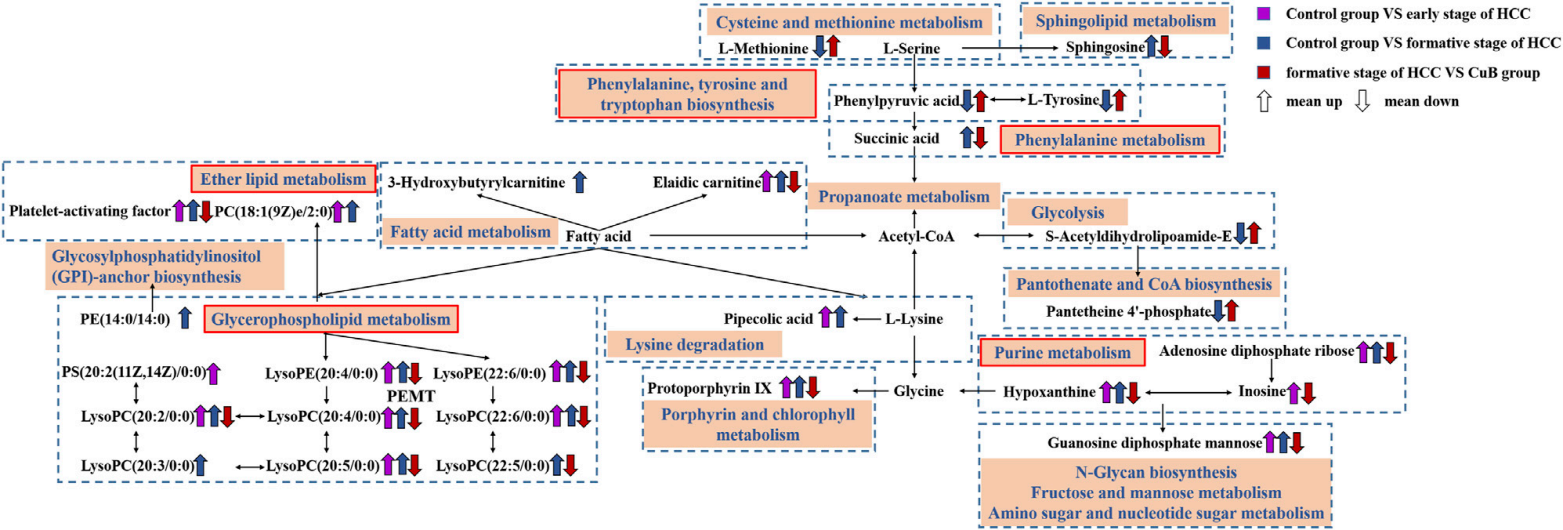
Metabolic network diagram. The rectangular boxes represent metabolic pathways. The purple arrows represent trends for Control group vs. early stage of HCC; The blue arrows represent trends for Control group vs. formative stage of HCC; The red arrows represent trends for formative stage of HCC vs. CuB group.

Based on biochemical knowledge, a metabolic network was formed from metabolites that were screened as differential metabolites in AKT/c-Met-induced HCC and CuB therapy, and this network was displayed using Cytoscape 3.8.2 software. The results are shown in Figure 5.

### Effect of Cucurbitacin B on AKT/mTORC1 signaling pathway

**FIGURE 6:**
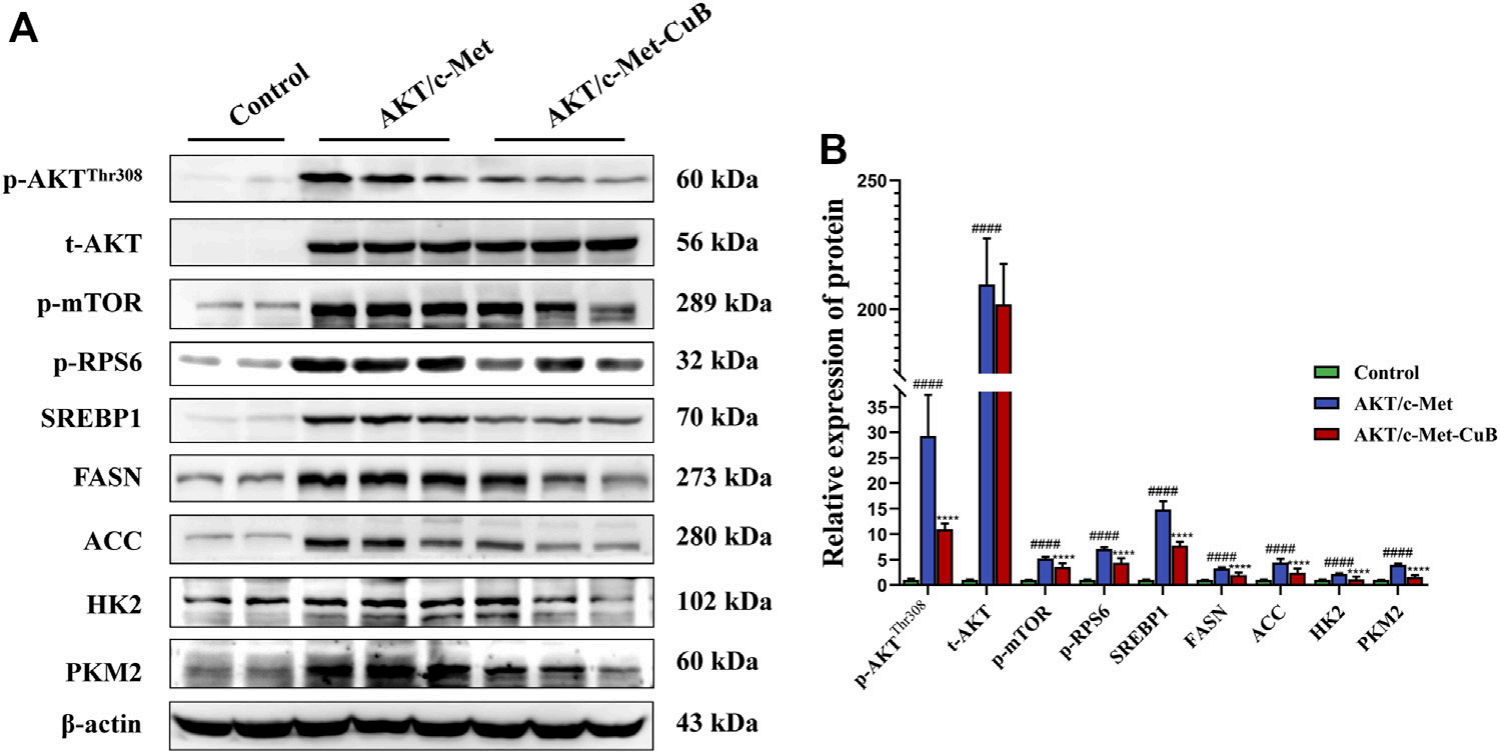
CuB inhibits the AKT/mTORC1 signal pathway in AKT/c-Met mice. **(A)** Western blotting analysis for different proteins; **(B)** Histograms depict the levels of protein expression. As an internal reference, β-actin was used as the housekeeping gene (^####^ *p* < 0.0001 vs. Control group; ****p < 0.0001 vs. AKT/c-Met group; quantitative data are the mean ± S.D. of the density values from the Western blotting experiment, *n* = 3, pounds and asterisks indicate statistical significance as determined by two-tailed unpaired *t*-test comparisons with two groups).

According to the results of CuB administration’s modulation of metabolic pathways, CuB predominantly affects lipid metabolism, amino acid metabolism, and glucose metabolism. Excessive lipogenesis and hepatocarcinogenesis have been linked to AKT/mTORC1 signaling activation, which upregulates FASN expression by boosting the transcriptional activity of SREBP1 (Calvisi et al., 2011). mTOR is a protein kinase that regulates protein synthesis and growth by mediating the effects of dietary amino acids (Wullschleger et al., 2006). The effect of CuB on the AKT/mTORC1 signaling pathway during HCC development was confirmed by examining related protein expression. CuB substantially decreased the expression levels of activated AKT and phosphorylated mTOR, including p-AKT^Thr308^, p-mTOR, and p-RPS6, in the liver lesions of AKT/c-Met-induced HCC mice, but total AKT levels remained unchanged. CuB therapy led to decreases in the protein expression levels of hepatic FASN and ACC, which are the key effectors of SREBP1-induced novo lipogenesis, since the lipogenic transcription regulator SREBP1 was downregulated at the protein level downstream of the AKT/mTORC1 cascade. The expression levels of HK2 and PKM2, critical proteins of glycolysis, were upregulated in AKT/c-Met group, indicating that glycolysis was triggered. CuB administration inhibited the protein expression of HK2 and PKM2. The results are shown in Figure 6 (*p* < 0.05).

## Discussion

HCC is one of the most prevalent malignant tumors in the world. In this study, we investigate the mechanisms of AKT/c-Met-induced hepatocellular carcinogenesis and the inhibitory effect of CuB in HCC by metabolomics techniques. The findings show that, AKT/c-Met-induced hepatocellular carcinogenesis is closely related to lipid metabolism, amino acid metabolism, and glucose metabolism. Furthermore, CuB improves lipid, amino acid, and glucose metabolism disorders by regulating the AKT/mTORC1 signaling pathway. In summary, this study for the first time elucidated the mechanisms of AKT/c-Met-induced hepatocellular carcinogenesis and the inhibitory effect of CuB in mice during the regulation of lipid metabolism, amino acid metabolism, and glucose metabolism *via* the AKT/mTORC1 pathway.

### Lipid metabolism for hepatocellular carcinogenesis and Cucurbitacin B treatment

In addition to being the primary raw materials and possible sources of energy for the body, lipids such as phospholipids, glycerol-lipids, and fatty acids are also significant signaling molecules that play a critical part in the physiological activities of the body (Lu et al., 2019). Tumor cell invasion, phospholipid metabolism, and inflammation are just some of the many biological processes that are affected by them.

Phospholipid metabolism includes sphingolipid, glycerophospholipid metabolism and the citrate cycle. Sphingolipid metabolism is closely linked to cell proliferation, differentiation, death, angiogenesis, inflammation, and tumorigenesis (Don and Rosen, 2009; Taniguchi and Okazaki, 2014). When compared to controls, the model group had higher concentrations of sphingosine, suggesting that the proliferation and migration of cancer cells may be accelerated due to an aberrant metabolism of sphingolipids. The back-regulation of sphingosine levels in the CuB group shown the possibility that CuB may intervene therapeutically by controlling sphingolipid metabolism. As important carriers of fatty acids, glycerophospholipids play a crucial role in maintaining basic cell structure and morphology, recognition, uptake and signal transduction of cellular signaling substances. The citrate cycle for fatty acids is required for the rapid multiplication of cancer cells to sustain β-oxidation. Succinic acid is an intermediate metabolite of the citrate cycle that is converted from α-KG to succinic acid by succinyl coenzyme A and then further converted to fumarate by succinic acid dehydrogenase (Wang and Huang, 2019). Inactivating mutations of succinate dehydrogenase can be found in tumors, leading to the accumulation of succinic acid in tumor cells, thus playing a role in promoting HCC tumor formation. It is possible that CuB delays HCC formation by regulating the citrate cycle, as seen by the dramatic drop in succinic acid levels after treatment. In this investigation, hepatocellular carcinogenesis and severe phospholipid metabolism disruption were tightly related. CuB may prevent the growth of tumors by controlling phospholipid metabolism in AKT/c-Met-induced HCC mice.

The altered cancer phenotype is distinguished by enhanced *de novo* lipogenesis. Improved *de novo* lipogenesis is characterized by increases in the activity and expression of a large number of lipogenic enzymes, such as FASN and ACC. During carcinogenesis, metabolic enzymes typically undergo mutations or amplification, which has been linked to the course of disease. These enzymes are highly expressed in numerous types of cancer, including HCC (Du et al., 2022). Fatty acid production is facilitated by cytoplasmic acetyl-CoA in cancer cells. Malonyl-CoA and acetyl-CoA can condense *via* FASN to produce fatty acid synthesis products, including palmitate. Patients with HCC who have elevated FASN have a poor prognosis. Nonalcoholic steatohepatitis is a disease vulnerable to HCC (Hu et al., 2016), which is accompanied by overexpression of FASN and severe steatosis. FASN overexpression and elevated activity promote the early start and development of this malignant phenotype. There is strong genetic and preclinical evidence that FASN plays a critical role in the steatosis and hepatocarcinogenesis of the liver. FASN deletion in hepatocytes reduces steatosis and proliferation, which is in accordance with this process (Li et al., 2016). Unlike adipogenesis, which promotes cell growth and survival in other malignancies, the constant expression of FASN appears to be critical for enhancing hepatocyte proliferation, which is amplified in AKT-driven hepatocarcinogenesis (Li et al., 2016). This study demonstrates that CuB suppresses AKT/c-Met in mouse hepatocellular carcinogenesis beginning with hepatic steatosis. The AKT/mTORC1 signaling pathway is a crucial regulatory mechanism for fatty acid production and regulates numerous cellular functions involved in cell survival, glycolipid metabolism, and tumor development. Activation of AKT/mTORC1 signaling promotes activation of SREBP1 and other fatty acid metabolism-related transcription factors of the DNL pathway, which in turn upregulate the key enzymes of hepatocyte fatty acid *ab initio* synthesis, FASN, ACC, etc., causing an increase in hepatocyte fatty acid *ab initio* synthesis (Laplante and Sabatini, 2009; Naik et al., 2013). And the AKT/mTORC1 signaling pathway plays an essential role in hepatocellular carcinogenesis, as demonstrated by the findings in this study. CuB may treat AKT/c-Met-induced HCC by blocking the AKT/mTORC1 signaling pathway and its downstream transcription factors and key enzymes involved in fatty acid synthesis.

### Other metabolisms for hepatocellular carcinogenesis and Cucurbitacin B treatment

Tumor cells need to utilize a large amount of amino acids for biomolecule synthesis. Tumor metabolic reprogramming also includes abnormal amino acid metabolism, which is primarily manifested by increased tumor cell uptake of specific nonessential amino acids, upregulation of transporter expression, and increased expression of metabolic enzymes for amino acid synthesis or catabolic processes (Wang and Huang, 2019). Since the majority of these amino acid metabolic processes take place in the liver, HCC development and incidence are ultimately accompanied by disruptions and changes in amino acid metabolic pathways. In the liver, amino acid metabolism and lipid metabolism are not independent processes but rather are tightly interconnected, coordinated, and interactive. Together with tyrosine, methionine is essential for the preservation of hepatic lipotropic activities (Chrétien and Li, 1967). Therefore, the changes in methionine levels also indicate to some extent a disturbance of lipid digestion in HCC, which also corresponds to the enhanced lipogenic activity during hepatocarcinogenesis (Yamashita et al., 2009). S-adenosylmethionine (SAM) is a derivative of methionine catalyzed by methionine adenosyltransferase, which can provide methyl donors for methylation modification of nucleic acids and histones, which in turn regulates the expression of oncogenes and oncogenes in tumor cells (Wang and Huang, 2019). Moreover, tyrosine is produced by the conversion of phenylalanine through phenylalanine, tyrosine and tryptophan biosynthesis. Previous research has demonstrated a substantial association between the malfunction of tyrosine metabolic pathways and liver cancer (Wang J. et al., 2022). Fumaric acid and acetoacetate, which are the end products of tyrosine metabolism, can be converted to acetyl-CoA, which is involved in lipid biosynthesis. This indicates that metabolite-mediated tyrosine metabolism and lipid metabolism interact and influence one another. CuB may enhance methionine metabolism, tyrosine metabolism, and lipid metabolism abnormalities by controlling methionine and tyrosine content and thereby treat AKT/c-Met-induced HCC, according to the experimental findings.

Glucoses are the most important and direct energy substances in the body, and their metabolic behavior is often greatly disturbed during the occurrence and development of HCC. The most representative related pathways include glycolysis. Glycolysis is one of the main ways for organisms to decompose sugar and glycogen to obtain energy and is also the most important way for tumor cells to obtain energy. Because glycolysis can be carried out without the participation of oxygen, cancer cells are more dependent on this “low-cost” energy supply during the runaway proliferation process. Even in aerobic settings, tumor cells convert glucose into lactate *via* glycolysis because of the energy requirements of fast cell multiplication. There is a term for this phenomenon: the Warburg effect (Heiden et al., 2009). HK-2 is a highly bound hexokinase isoform that is commonly overexpressed in human cancers involving the activation of aerobic glycolysis, including HCC. Pyruvate kinase (PK) is a critical rate-limiting enzyme involved in the final step of glycolysis (Zhang et al., 2019). In this study, tumor cells supported energy and anabolism by upregulating HK2 and PKM2 expression to meet the metabolic requirements for abnormal proliferation. The downregulation of HK2 and PKM2 expression after CuB administration suggests that the rapid proliferation of HCC cells can be controlled by controlling glycolysis, thereby preventing the formation of HCC. S-Acetyldihydrolipoamide-E was considerably upregulated following CuB administration, suggesting that the formation of HCC could be prevented by controlling glycolysis. In addition, succinic acid and guanosine diphosphate mannose were involved in the citrate cycle and fructose and mannose metabolism, and the levels were significantly upregulated in HCC. The levels were significantly back-regulated after CuB administration, suggesting that CuB may inhibit the rapid proliferation of HCC cells through the regulation of glucose metabolism, thus inhibiting the development of HCC. Patients suffering from type 2 diabetes mellitus exhibit significant hyperglycemia and decreased insulin secretion (Clark et al., 2001). A related study showed that CuB decreased body weight and blood glucose concentrations in mice with type 2 diabetes mellitus model (Dwijayanti et al., 2020). It also increased serum insulin concentration and hepatic insulin receptor mRNA levels and enhanced insulin secretion. This suggests that CuB has a hypoglycemic effect in hyperglycemia.

Metabolism of acylcarnitine is a crucial link in a complex metabolic network that regulates lipid and glucose homeostasis (Qu et al., 2016). The accumulation of acetyl-CoA metabolic intermediates in mitochondria might result in undesirable side effects. Acylcarnitine inhibits the activity of carnitine palmitoyltransferase I (CPTI) by converting to malonyl-CoA, thereby reducing fatty acid oxidation and eliminating adverse effects (Casals et al., 2016). In addition, acylcarnitine is engaged in many physiological processes, including the synthesis of ketone bodies and the peroxidation of fatty acids. When the glucagon/insulin ratio decreases, acylcarnitine stimulates pyruvate dehydrogenase activity, which enhances pyruvate oxidation and the aerobic oxidation of glucose (Seiler et al., 2015). According to the findings, the levels of elaidic carnitine and 3-Hydroxybutyrylcarnitine were significantly upregulated in HCC. CuB may regulate acylcarnitine metabolism by downregulating the level of acylcarnitine, thereby balancing lipid and glucose metabolism for the treatment of AKT/c-Met-induced HCC.

## Conclusion

In this work, a mouse model of HCC generated by AKT/c-Met was utilized. To evaluate the metabolomic approach of hepatocellular carcinogenesis, compared to the control group, 26 predifferential and 36 formative-differential metabolites were discovered in blood serum samples, and 13 formative-differential metabolites were discovered in liver samples. The primary metabolic routes were lipid, amino acid, and glucose metabolism. CuB is a naturally occurring chemical with anticancer potential. On the basis of metabolomics analyses, 33 and 11 possible biomarkers were discovered in serum and liver samples, respectively, most of which were associated with lipid, amino acid, and glucose metabolism. The AKT/mTORC1 signaling pathway may be regulated by CuB by rearranging lipid metabolism, amino acid metabolism, and glucose metabolism, which could limit tumor growth. CuB improves lipid, amino acid, and glucose metabolism disorders by regulating the AKT/mTORC1 signaling pathway, thereby inhibiting further formation and progression of HCC and opening up a new avenue for the clinical treatment of HCC by inhibiting the activities of lipids, amino acids, and glucose. These findings could provide a metabolic basis for the early detection, treatment, and prognosis of HCC patients, as well as the therapeutic application of CuB in HCC.

## Data Availability

Data availability statement was changed that, the original contributions presented in the study are included in the article/Supplementary Material, further inquiries can be directed to the corresponding authors.
