# Supplementary material for: Metabolomics profiling of AKT/c-Met-induced hepatocellular carcinogenesis and the inhibitory effect of Cucurbitacin B in mice
Source: Front Pharmacol. 2022 Nov 23;13:1009767. doi: 10.3389/fphar.2022.1009767 (PMC9728611; doi:10.3389/fphar.2022.1009767)
Supplement: Supplementary file 1 [file DataSheet1.docx]

**Metabolomics Profiling of AKT/c-Met-induced** **Hepatocellular Carcinogenesis and the Inhibitory Effect of Cucurbitacin B in Mice**

Xiangyu Ji ^1^, Xin Chen ^1^, Lei Sheng ^1^, Dongjie Deng ^1^, Qi Wang ^1^, Yan Meng ^1^, Zhenpeng Qiu ^1^, Baohui Zhang ^1^, Guohua Zheng ^1,2*^ and Junjie Hu ^1*^

^1^ College of Pharmacy, Hubei University of Chinese Medicine, Wuhan, Hubei, People’s Republic of China

^2^ Key Laboratory of Chinese Medicine Resource and Compound Prescription, Ministry of Education, Hubei University of Chinese Medicine, Wuhan, Hubei, People’s Republic of China

**Correspondence:** Junjie Hu ([hero0712@163.com](mailto:hero0712@163.com)) or Guohua Zheng ([zgh1227@sina.com](mailto:zgh1227@sina.com))

These authors contributed equally: Xiangyu Ji, Xin Chen.

**Supplementary Tables**

**Table S1** **List of the primary antibodies used for Western blotting (WB) and Immunohistochemistry (IHC) analysis.**

| **Protein** | **Antibody (and catalog number)** | **Application** |
| --- | --- | --- |
| Phospho-AKT^Thr308^ | Rabbit monoclonal (13038) | WB † |
| Total-AKT | Rabbit monoclonal (4691) | WB † |
| Phospho-mTOR | Rabbit monoclonal (ab109268) | WB ^ |
| Phospho-RPS6 | Rabbit monoclonal (4858) | WB † |
| SREBP1 | Rabbit polyclonal (GB11524) | WB ^@^ |
| FASN | Rabbit monoclonal (3180) | WB † |
| ACC | Rabbit monoclonal (3676) | WB † |
| HK2 | Rabbit monoclonal (2867) | WB † |
| PKM2 | Rabbit monoclonal (4053) | WB † |
| β-actin | Mouse monoclonal (A1978) | WB ^ |
| Ki67 | Rabbit monoclonal (ab16667) | IHC ^ |
| PCNA | Rabbit monoclonal (#13110) | IHC † |

† Provided by Cell Signaling Technology Inc. (Danvers, MA).

^ Provided by Abcam (Cambridge, MA).

^@^ Provided by ABclonal (Wuhan, China).

**Table S2 Repeatability and stability results for HCC formation induced by AKT/c-Met co-expression and CuB treatment in serum.**

|  | **ESI (+)** | | | | **ESI (-)** | | | |
| --- | --- | --- | --- | --- | --- | --- | --- | --- |
|  | 6 QC sample | | All QC sample | | 6 QC sample | | All QC sample | |
|  | RT | Area | RT | Area | RT | Area | RT | Area |
| 1 | 8.609 | 300072.23 | 8.612 | 398839.7 | 8.644 | 23174.81 | 8.631 | 26251.20 |
| 2 | 8.653 | 266889.13 | 8.618 | 344493.87 | 8.626 | 27155.33 | 8.644 | 19091.41 |
| 3 | 8.643 | 279007.05 | 8.627 | 259509.72 | 8.657 | 26222.89 | 8.669 | 19960.87 |
| 4 | 8.623 | 260572.25 | 8.609 | 350175.06 | 8.631 | 26501.89 | 8.672 | 20630.16 |
| 5 | 8.618 | 248379.56 | 8.597 | 354321.37 | 8.638 | 28944.60 | 8.687 | 17628.04 |
| 6 | 8.627 | 259509.72 | 8.597 | 409944.67 | 8.633 | 25170.66 | 8.673 | 19768.85 |
|  |  |  |  |  |  |  |  |  |
| Average | 8.6288 | 269071.6567 | 8.610 | 352880.7317 | 8.6382 | 26195.03 | 8.6627 | 20555.0883 |
| Standard deviation | 0.016327 | 18200.793506 | 0.011798 | 53228.566005 | 0.011089 | 1936.404568 | 0.020868 | 2970.944504 |
| RSD (%) | 0.1892 | 6.7643 | 0.137 | 15.084 | 0.1284 | 7.3923 | 0.2409 | 14.4536 |

**Table S3 Repeatability and stability results for HCC formation induced by AKT/c-Met co-expression and CuB treatment in liver.**

|  | **ESI (+)** | | | | **ESI (-)** | | | |
| --- | --- | --- | --- | --- | --- | --- | --- | --- |
|  | 6 QC sample | | All QC sample | | 6 QC sample | | All QC sample | |
|  | RT | Area | RT | Area | RT | Area | RT | Area |
| 1 | 8.601 | 1452188.82 | 8.605 | 1530488.09 | 8.564 | 126210.56 | 8.553 | 131970.45 |
| 2 | 8.605 | 1436913.01 | 8.595 | 1595843.5 | 8.562 | 128802.6 | 8.556 | 127050.09 |
| 3 | 8.605 | 1530488.09 | 8.606 | 948354.26 | 8.553 | 131970.45 | 8.575 | 141846.64 |
| 4 | 8.631 | 1688123.59 | 8.607 | 900834.05 | 8.567 | 123523.86 | 8.564 | 126210.56 |
| 5 | 8.595 | 1595843.5 | 8.605 | 1436913.01 | 8.555 | 123196.17 | 8.568 | 117818.1 |
| 6 | 8.631 | 1281622.24 | 8.597 | 1077534.95 | 8.556 | 127050.09 | 8.562 | 128802.6 |
| 7 |  |  | 8.601 | 1452188.82 |  |  | 8.567 | 123523.86 |
| 8 |  |  | 8.631 | 1281622.24 |  |  | 8.553 | 139074.65 |
| 9 |  |  | 8.631 | 1688123.59 |  |  | 8.555 | 123196.17 |
|  |  |  |  |  |  |  |  |  |
| Average | 8.6113 | 1497529.875 | 8.6087 | 1323544.7233 | 8.5595 | 126792.2883 | 8.5614 | 128832.5689 |
| Standard deviation | 0.015667 | 141013.545049 | 0.013304 | 287450.452935 | 0.005612 | 3312.831769 | 0.007732 | 7709.049369 |
| RSD (%) | 0.1819 | 9.4164 | 0.1545 | 21.7182 | 0.0656 | 2.6128 | 0.0903 | 5.9838 |

| **Table S4 OPLS-DA analysis and the permutation test parameters in serum and liver of Control, Model and CuB group.** | | | | | | | | | | | | | | | | | | | |  |
| --- | --- | --- | --- | --- | --- | --- | --- | --- | --- | --- | --- | --- | --- | --- | --- | --- | --- | --- | --- | --- |
| **Groups** | | | **Type** | | **A** | | **R²X(cum)** | | **R²Y(cum)** | | **Q²(cum)** | | **Ion Mode** | | **R²intercept** | | **Q²intercept** | | **Position** | |
|  |  |  |  |  |  |  |  |  |  |  |  |  |  |  |  |  |  |  |  |  |
| Control vs Model 3W | | | OPLS-DA | | 4 | | 0.811 | | 0.694 | | 0.250 | | Positive | | 0.448 | | -0.697 | | serum | |
| Control vs Model 6W | | | OPLS-DA | | 4 | | 0.872 | | 0.980 | | 0.836 | | Positive | | 0.824 | | -0.867 | | serum | |
| Control vs Model 6W | | | OPLS-DA | | 4 | | 0.736 | | 0.998 | | 0.971 | | Positive | | 0.875 | | -0.563 | | liver | |
| Control vs Model 3W | | | OPLS-DA | | 3 | | 0.822 | | 0.663 | | 0.454 | | Negative | | 0.310 | | -0.491 | | serum | |
| Control vs Model 6W | | | OPLS-DA | | 4 | | 0.893 | | 0.971 | | 0.646 | | Negative | | 0.714 | | -0.657 | | serum | |
| Control vs Model 6W | | | OPLS-DA | | 4 | | 0.687 | | 0.983 | | 0.916 | | Negative | | 0.703 | | -0.828 | | liver | |
| Control vs Model 3W vs Model 6W vs CuB | | | OPLS-DA | | 4 | | 0.826 | | 0.575 | | 0.484 | | Positive | | 0.147 | | -0.254 | | serum | |
| Control vs Model 6W vs CuB | | | OPLS-DA | | 5 | | 0.640 | | 0.494 | | 0.443 | | Positive | | 0.312 | | -0.489 | | liver | |
| Control vs Model 3W vs Model 6W vs CuB | | | OPLS-DA | | 4 | | 0.836 | | 0.594 | | 0.467 | | Negative | | 0.169 | | -0.254 | | serum | |
| Control vs Model 6W vs CuB | | | OPLS-DA | | 3 | | 0.589 | | 0.614 | | 0.463 | | Negative | | 0.267 | | -0.522 | | liver | |
|  |  |  | |  | |  | |  | |  | |  | |  | |  | |  | |  |

**Supplementary Figures**

**
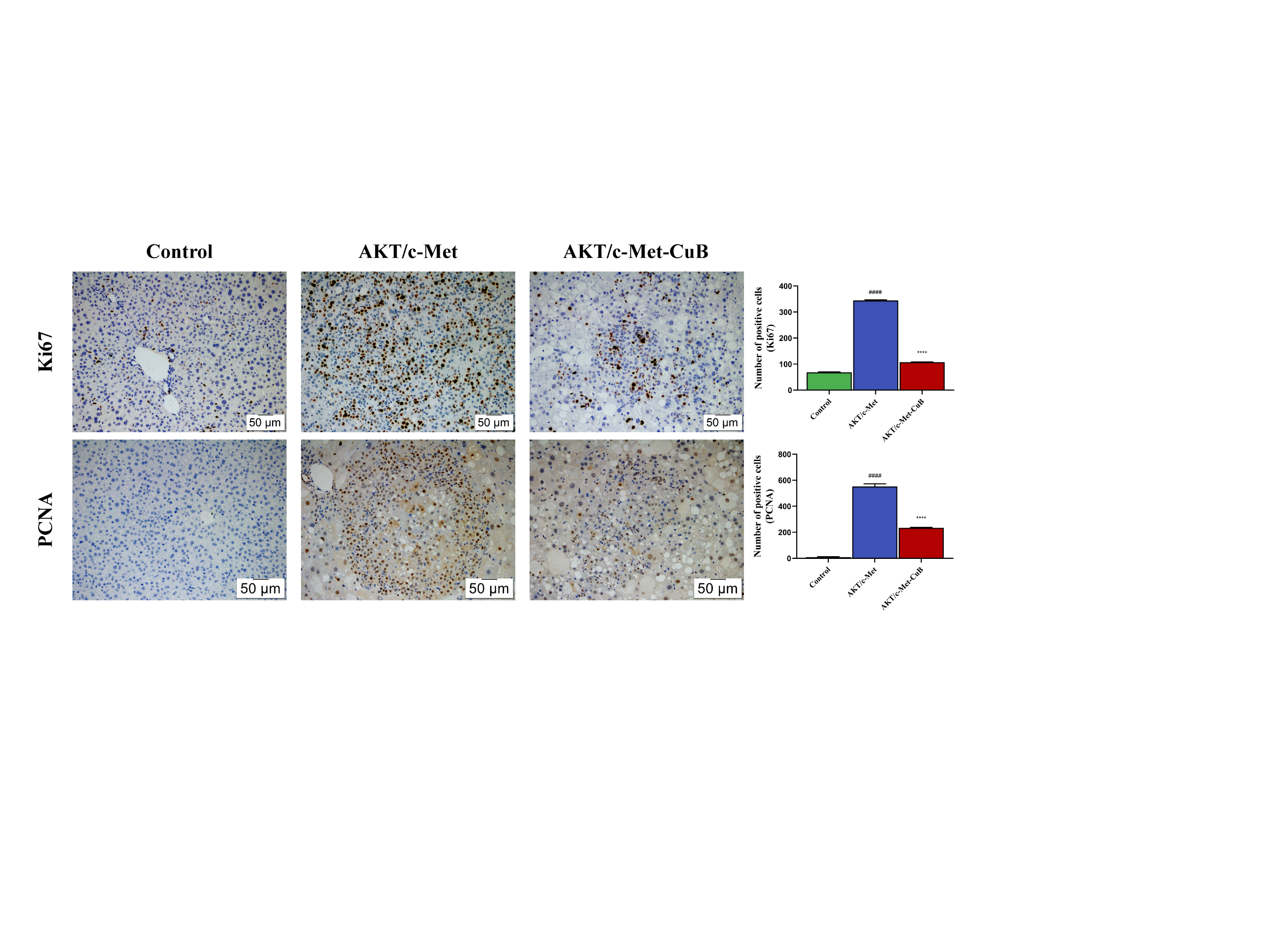
**

**Figure S1. Immunohistochemical staining of Ki67, PCNA and histogram of positive cell counts.** CuB inhibits Ki67 and PCNA accumulation in the livers of the AKT/c-Met mice. Original magnification: 200×; scale bar: 50 μm. (Mean ± S.D., n = 3. ^####^*P*<0.0001 VS Control group; ^⁎⁎⁎⁎^*P*<0.0001 VS AKT/c-Met group, pounds and asterisks indicate statistical significance as determined by two-tailed unpaired t-test comparisons with two groups).

**Figure S2. TIC stacking diagram of each group of serum in positive ion mode. A:** Control group; **B:** early stage of HCC; **C:** formative stage of HCC.

**Figure S3. TIC stacking diagram of each group of serum in negative ion mode. A:** Control group; **B:** early stage of HCC; **C:** formative stage of HCC.

**Figure S4. TIC stacking diagram of each group of liver in positive and negative ion mode. A, B:** Control group; **C, D:** formative stage of HCC.

 **Figure S5. PCA and PLS-DA Analysis. A:** PCA score of each group of serum in positive and negative ion modes; **B:** PLS-DA score of each group of serum in positive and negative ion modes; **C:** PLS-DA score of each group of liver in positive and negative ion modes.

**Positive mode**


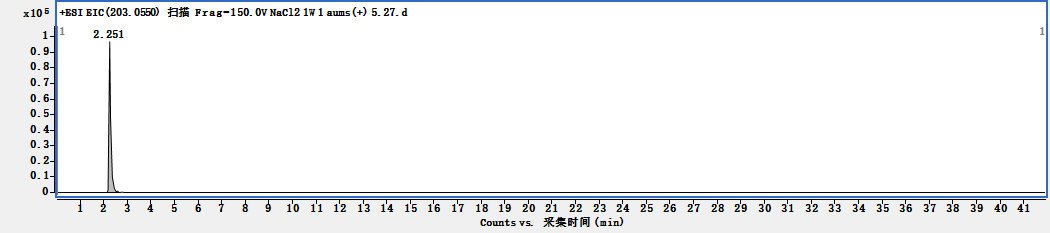

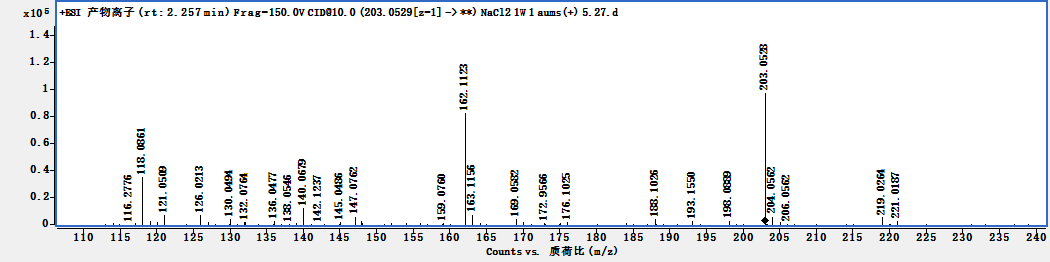

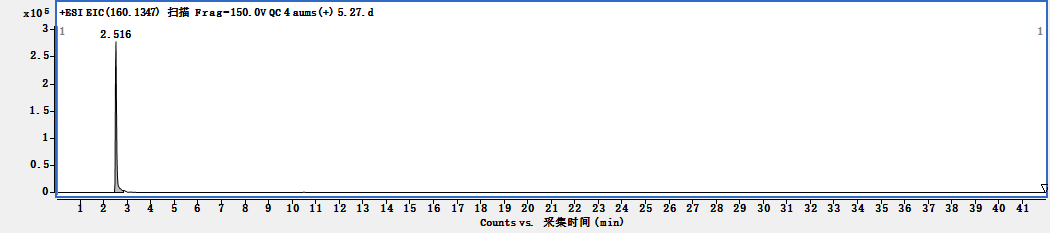

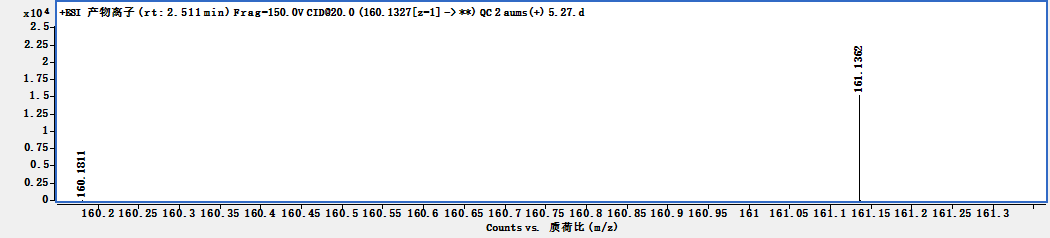

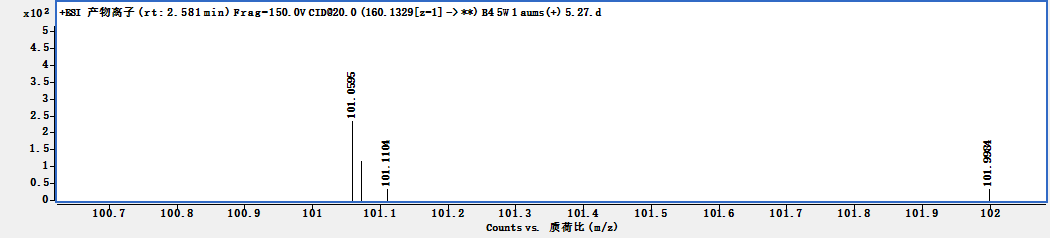

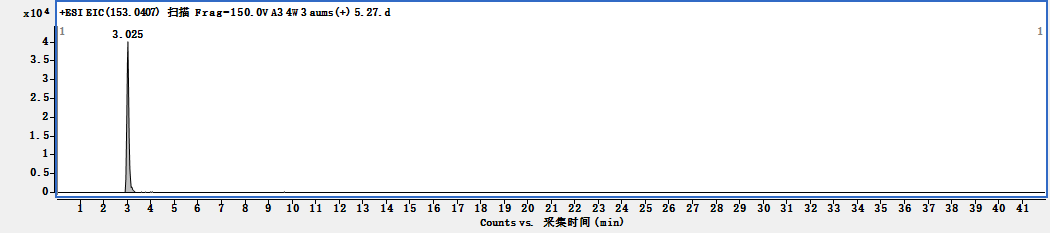

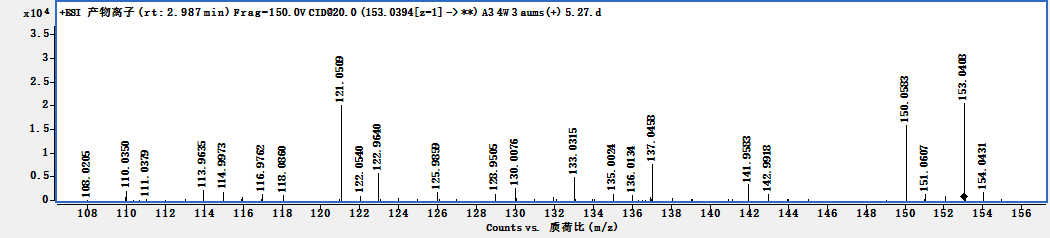

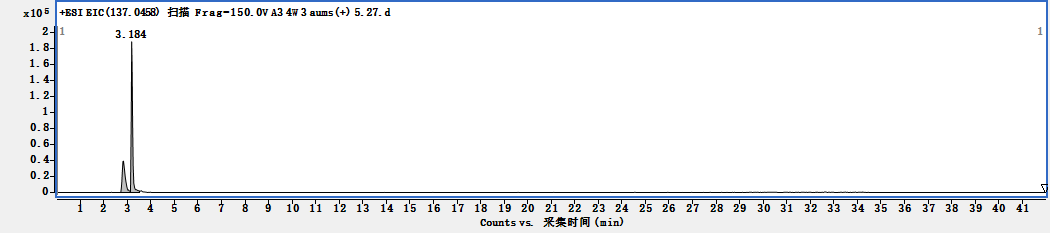

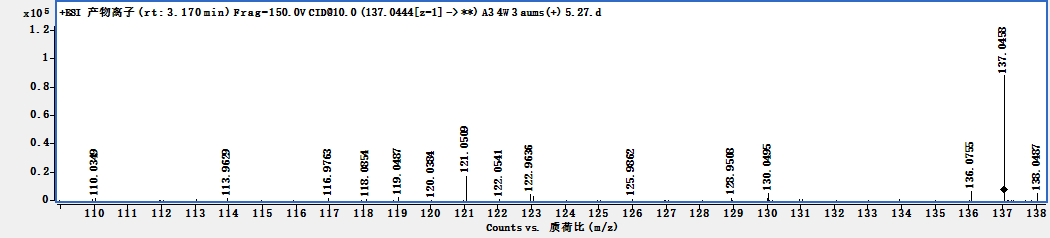

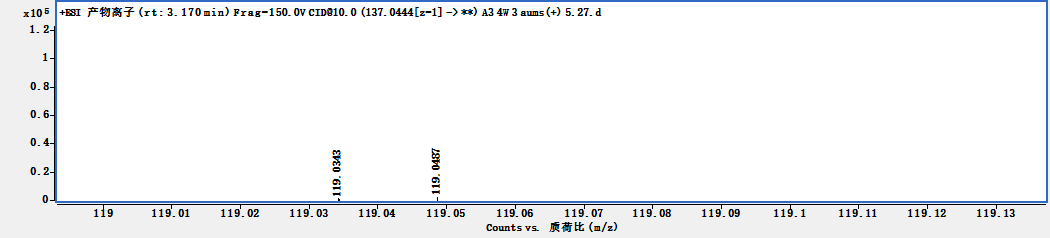

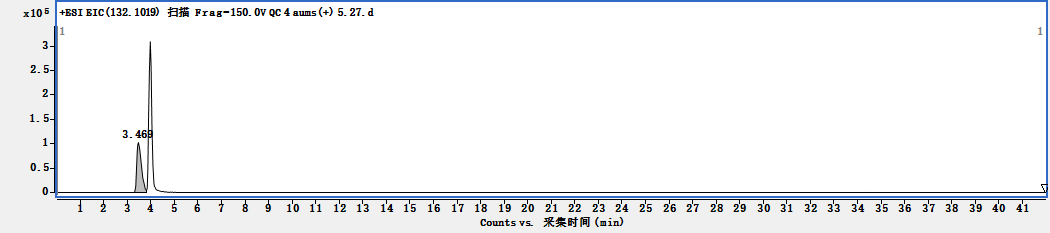

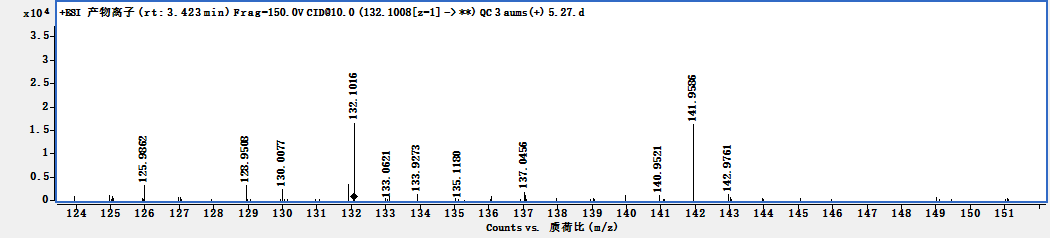

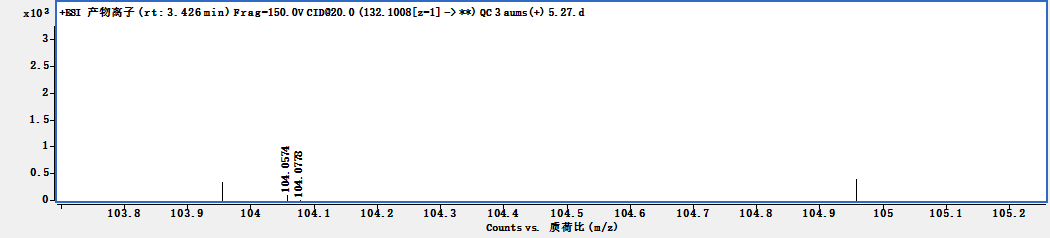

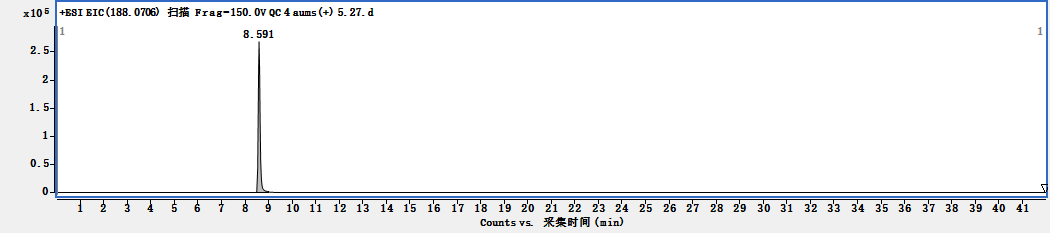

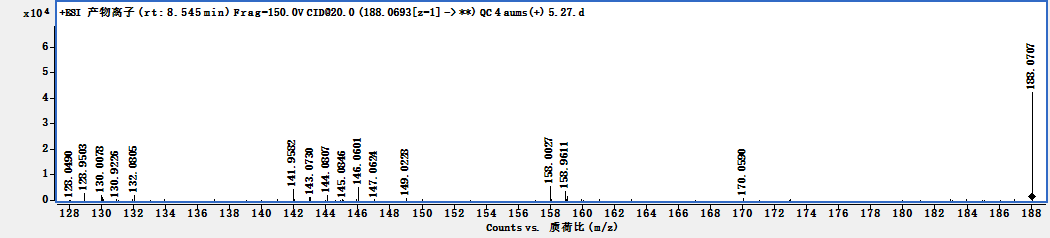

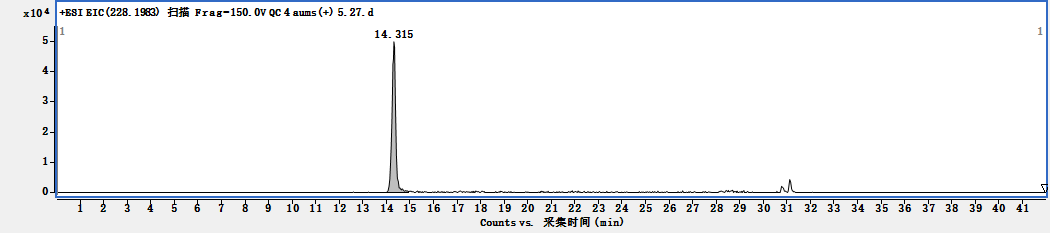

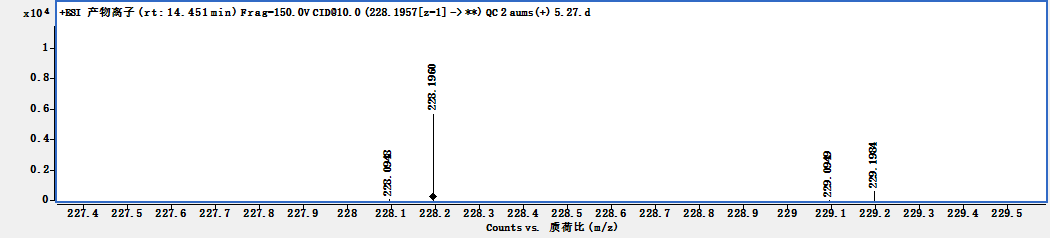

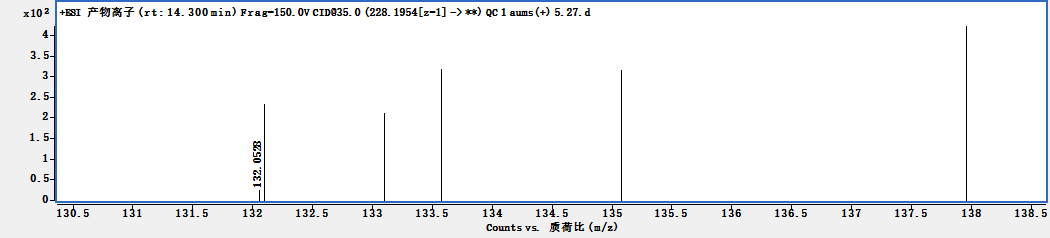

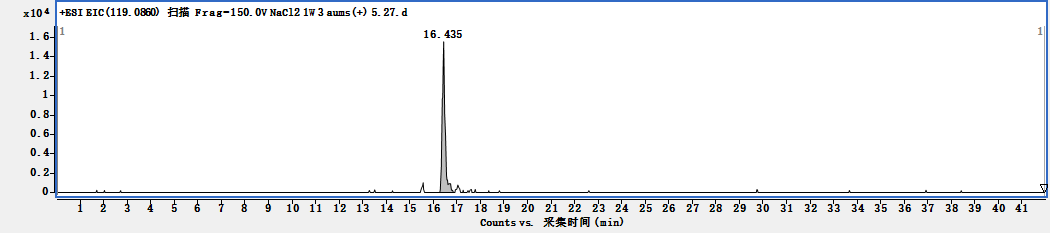

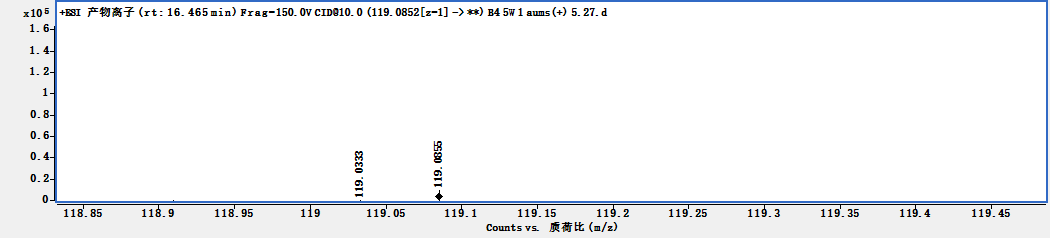

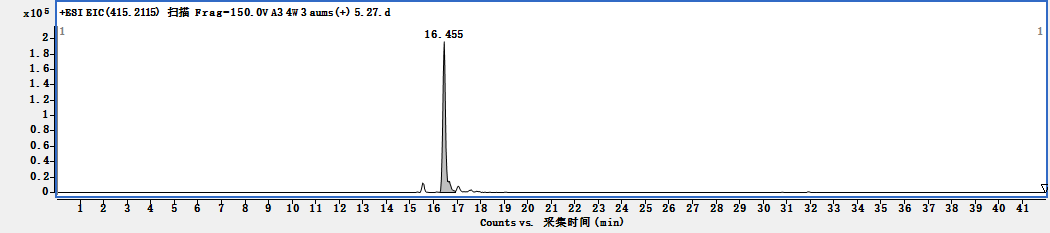

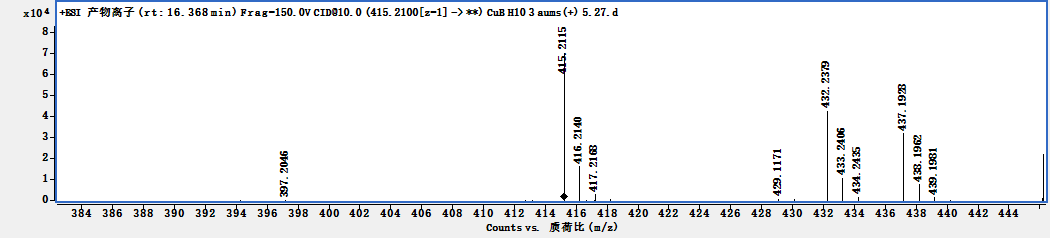

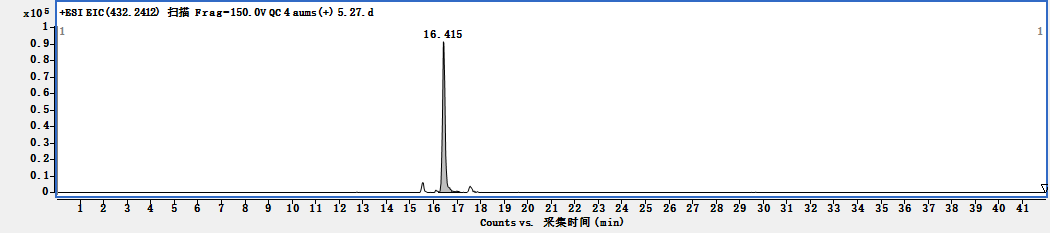

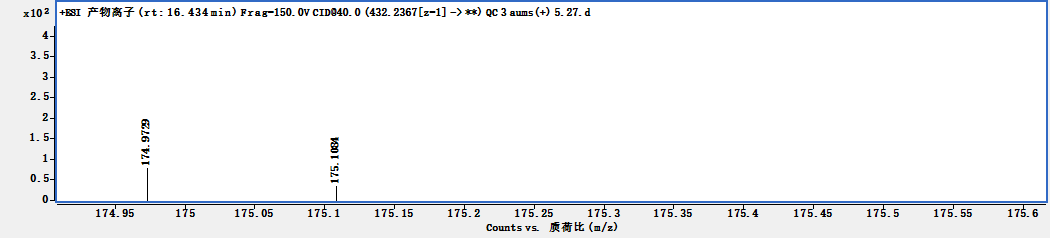

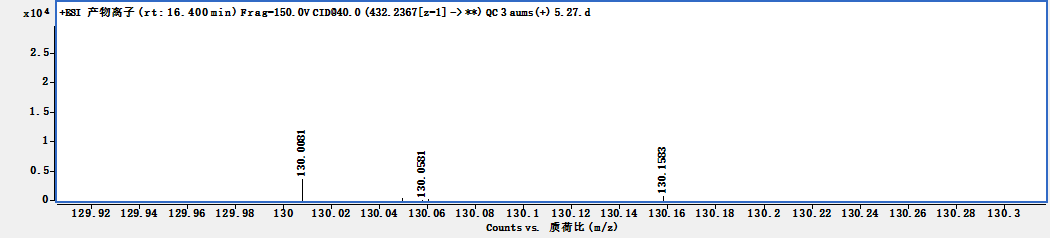

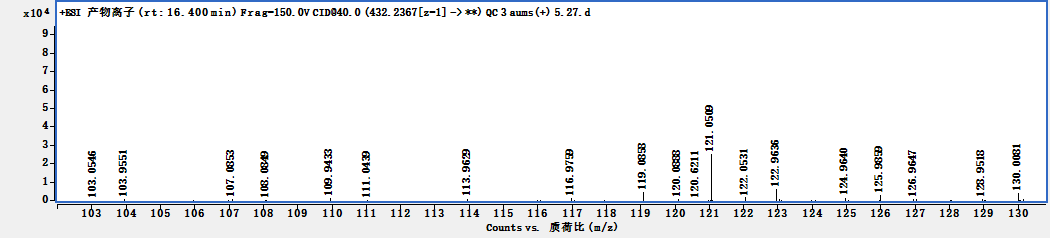

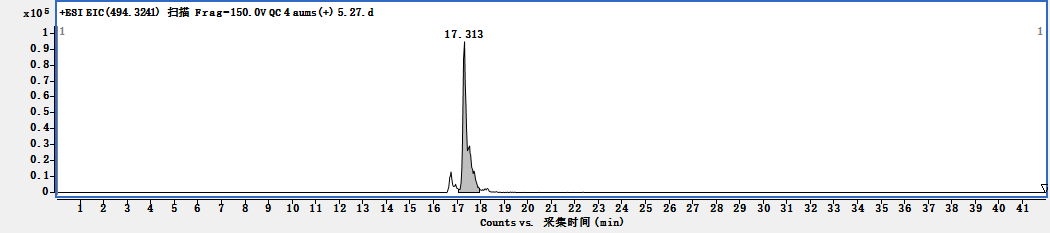

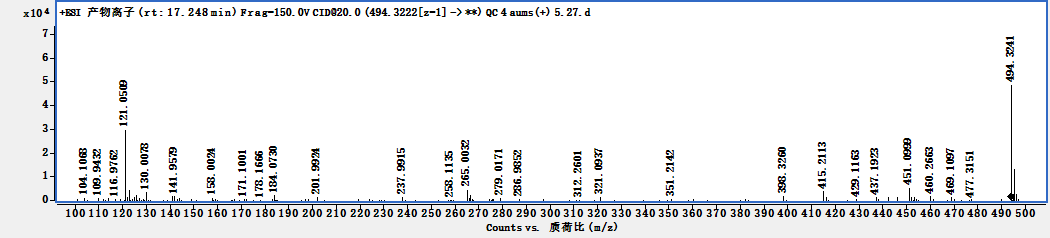

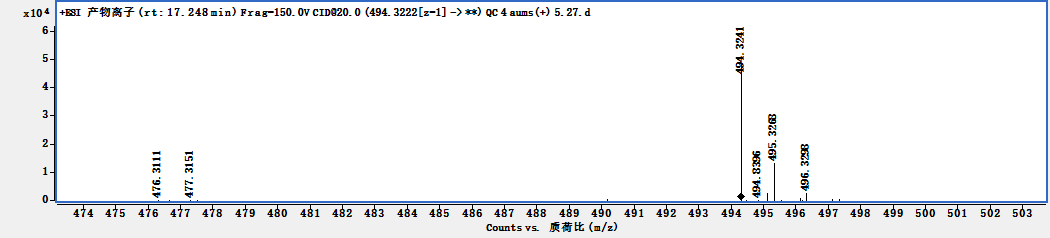

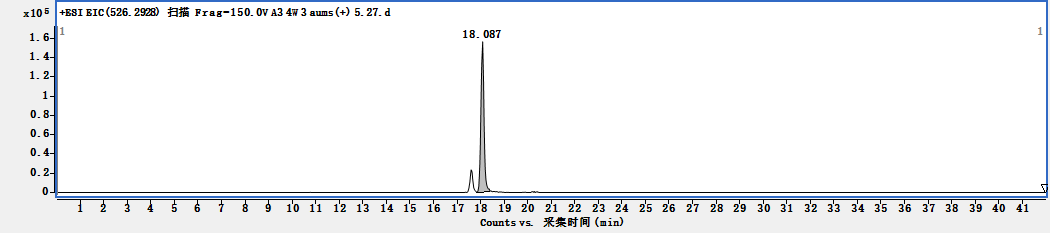

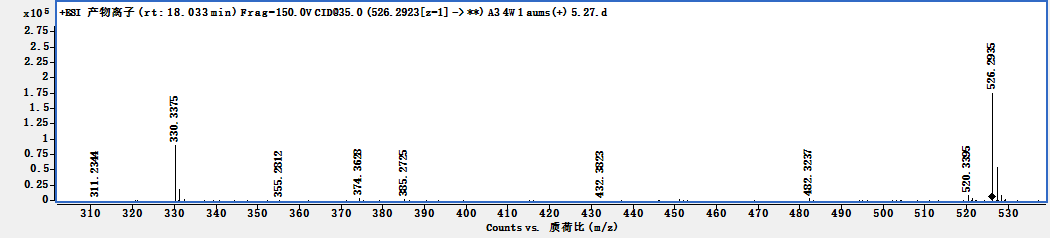

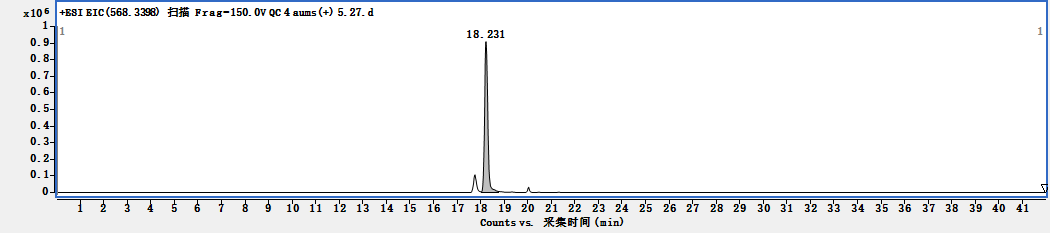

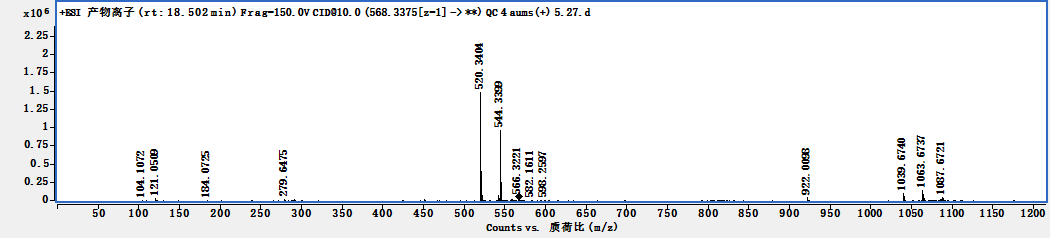

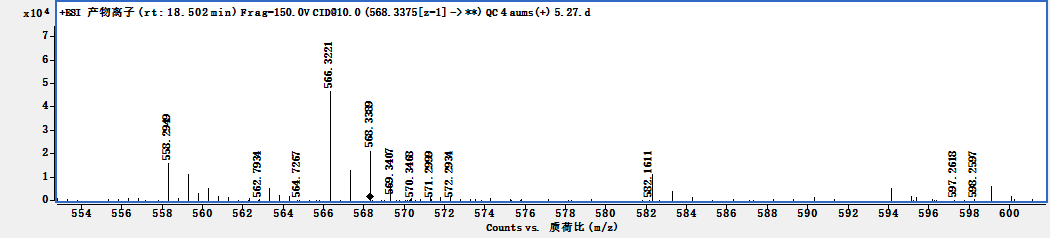

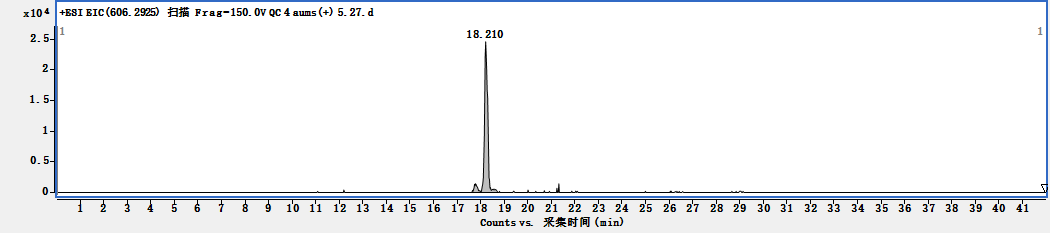

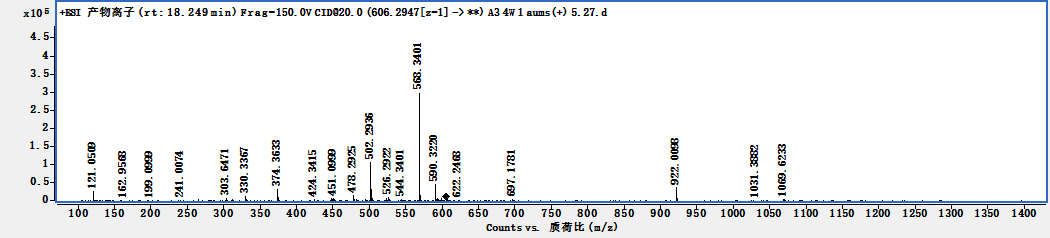

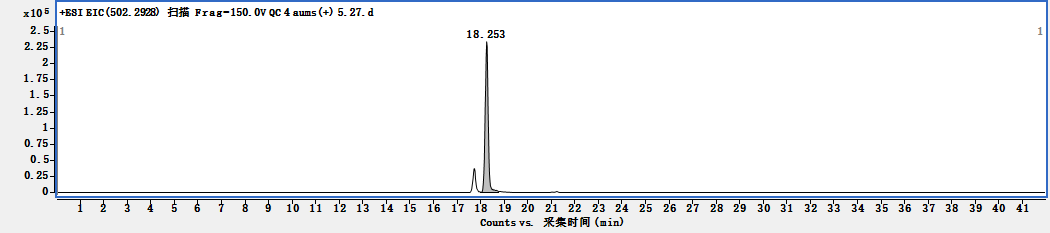

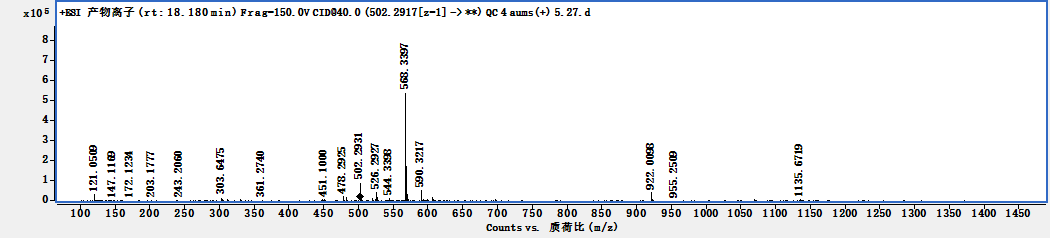

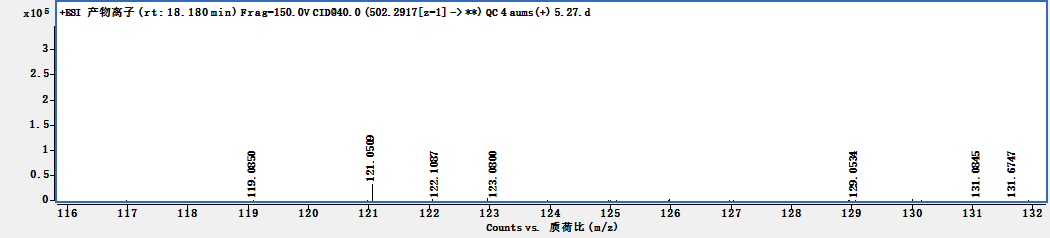

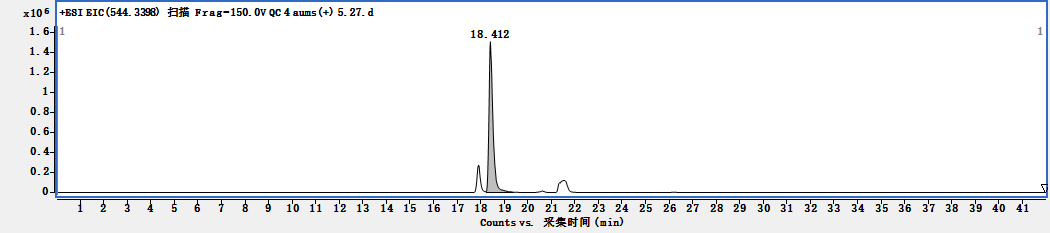

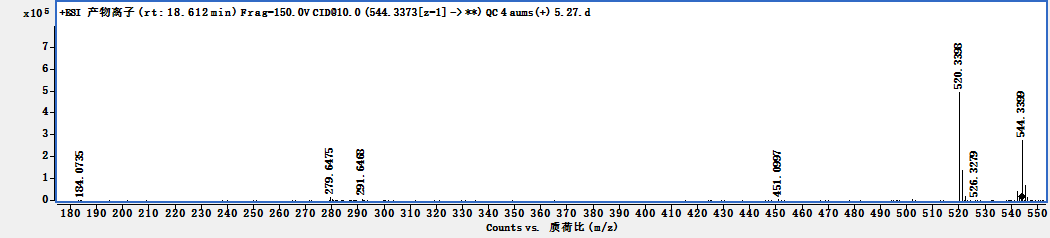

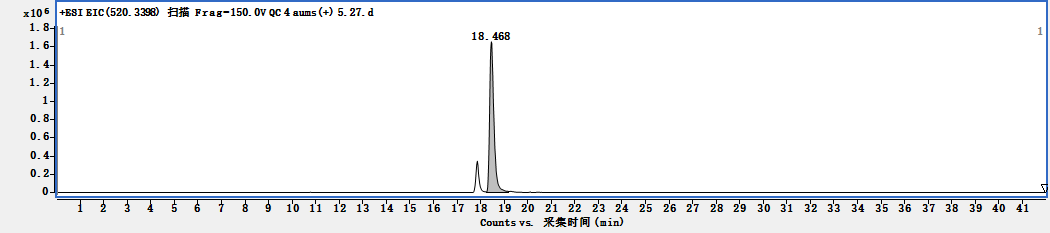

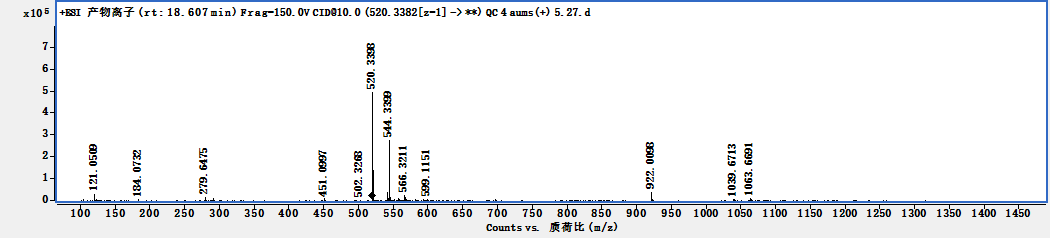

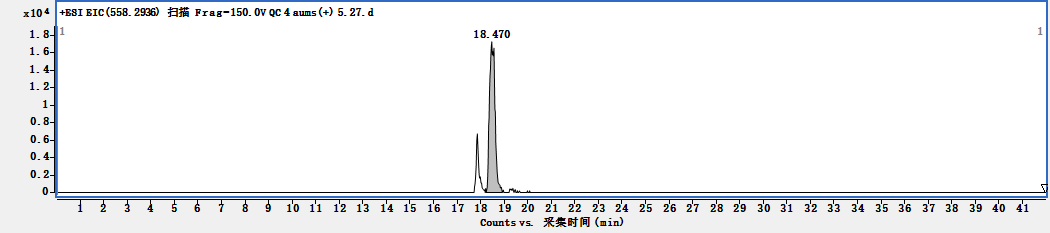

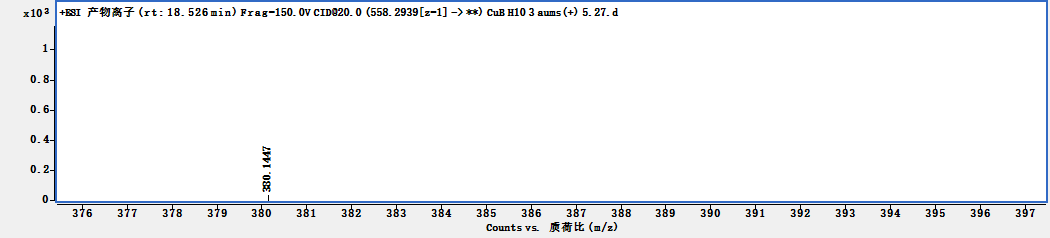

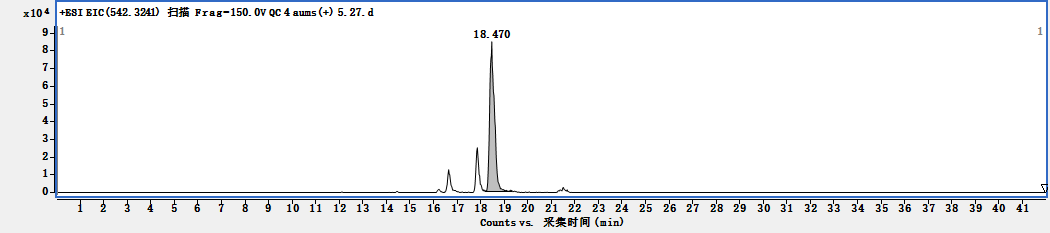

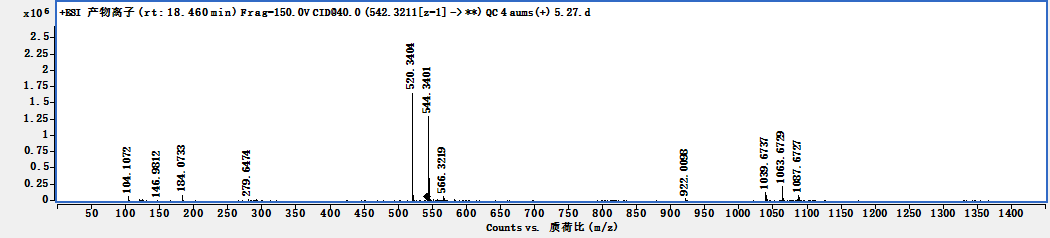

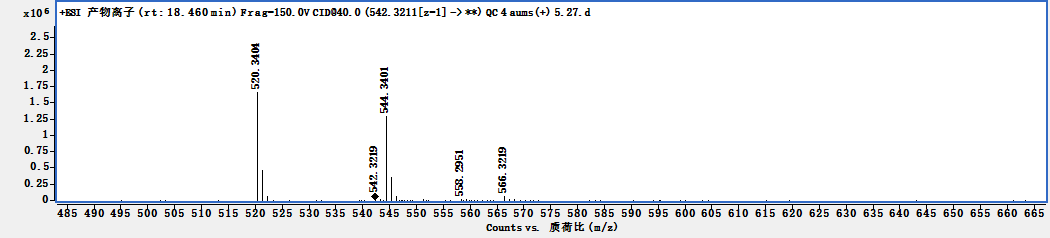

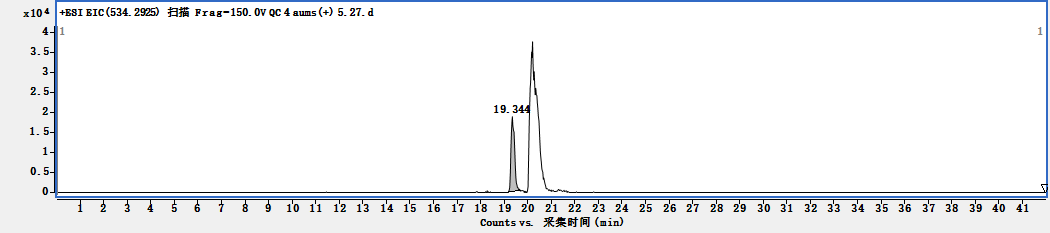

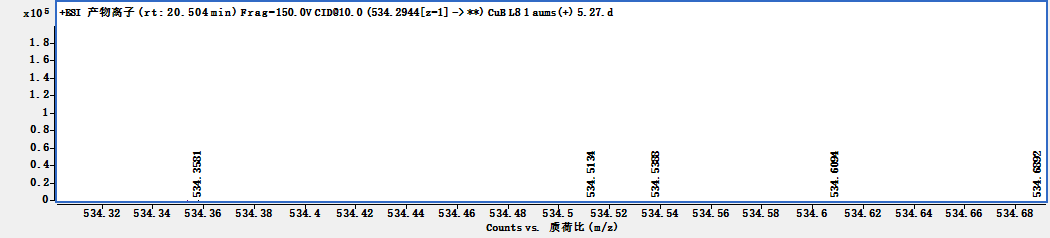

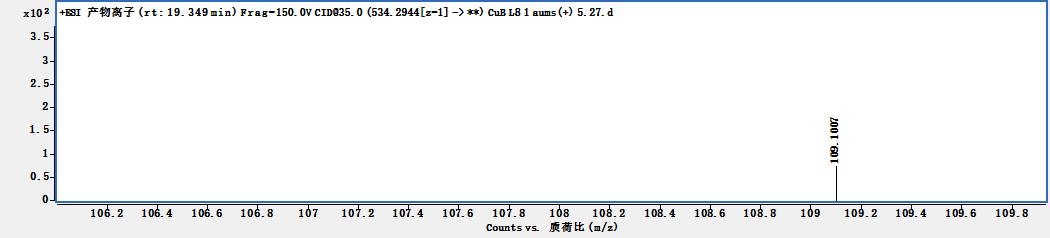

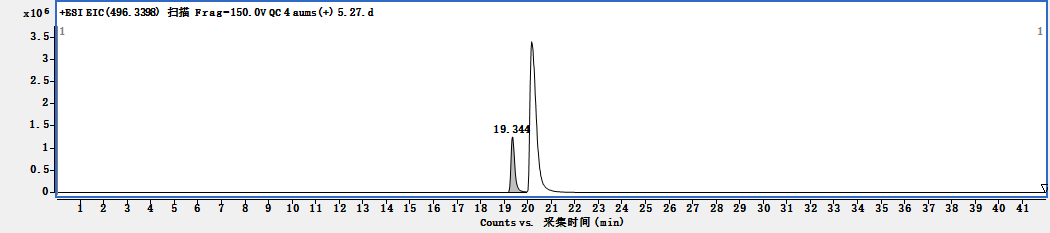

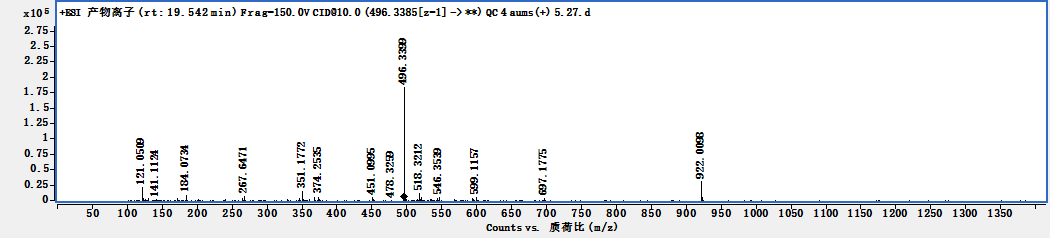

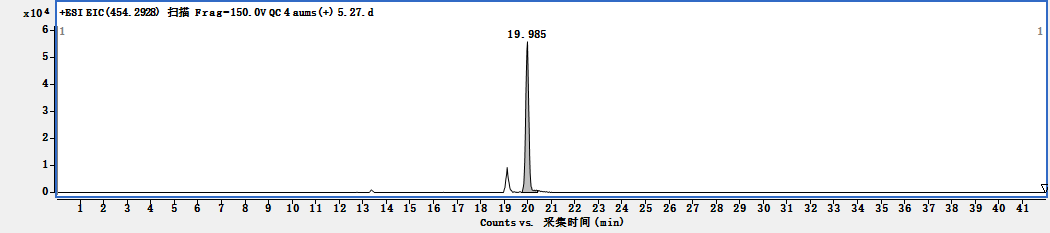

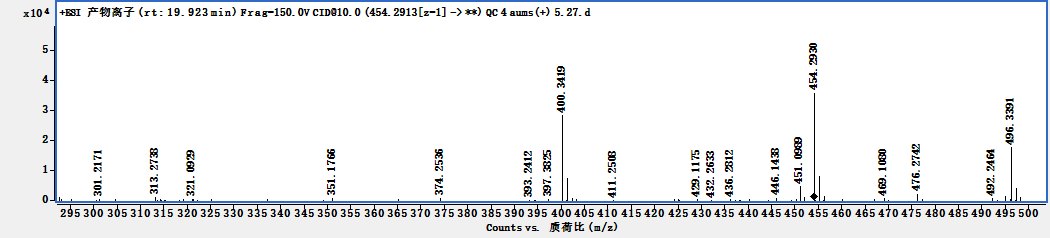

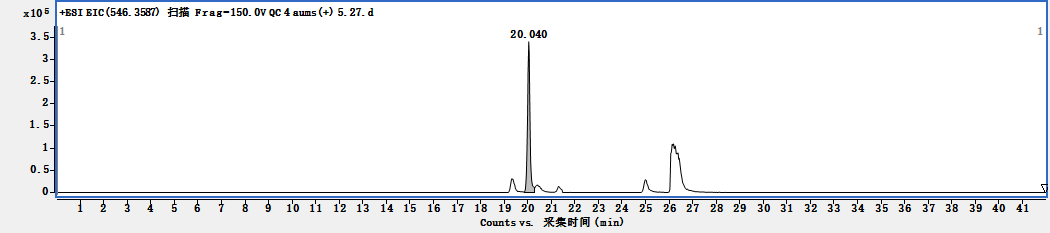

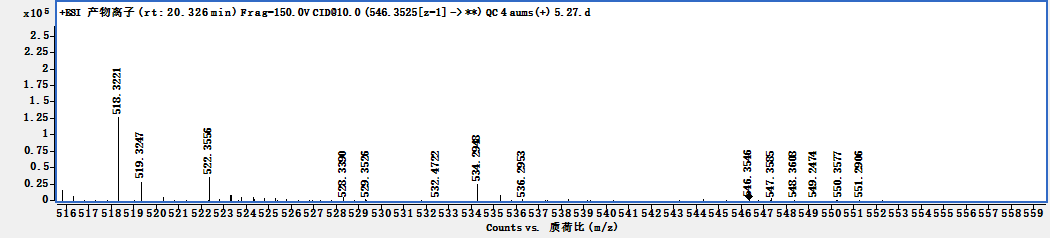

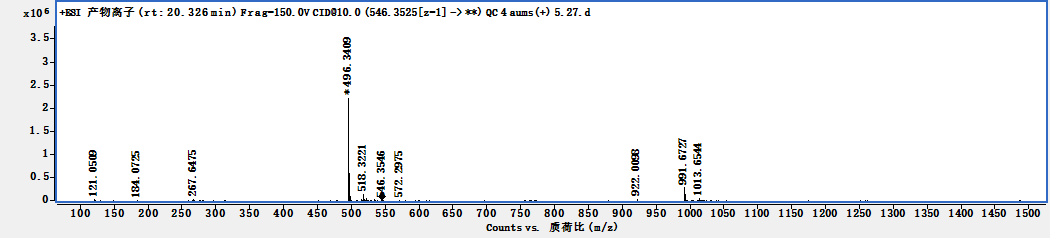

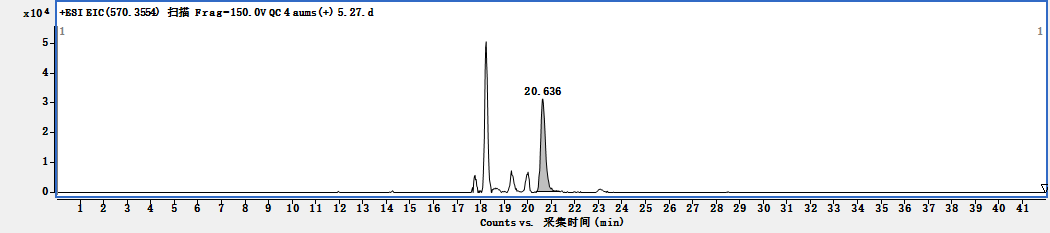

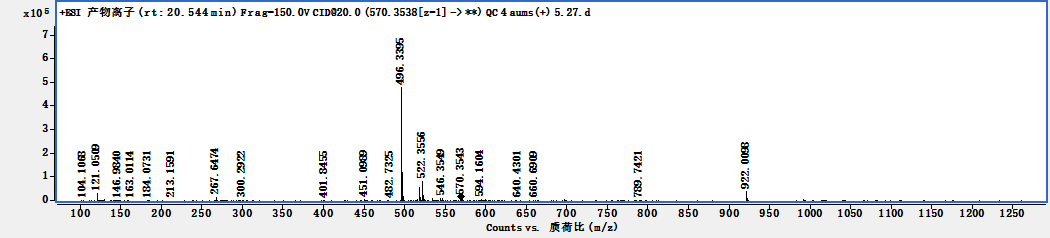

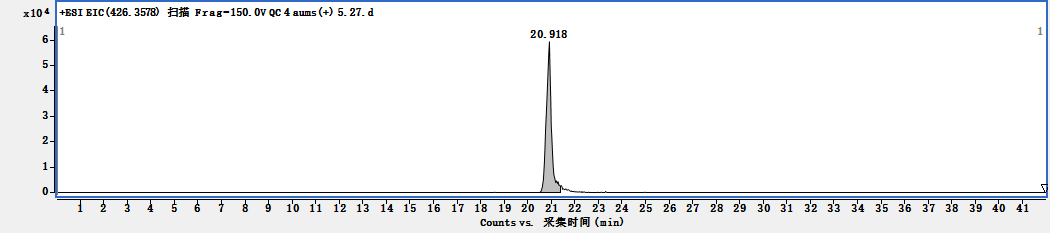

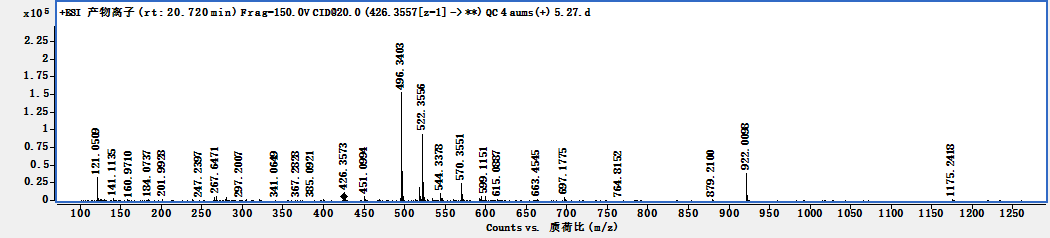

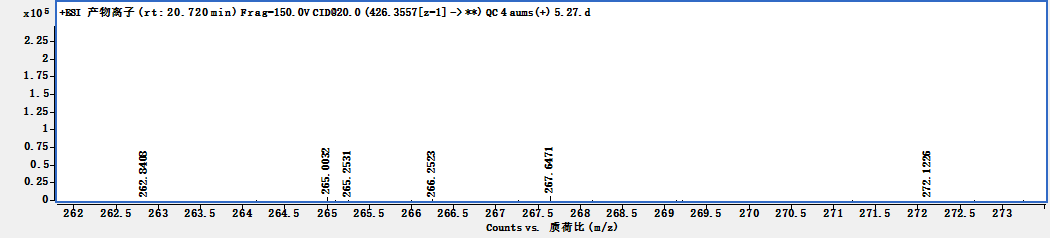

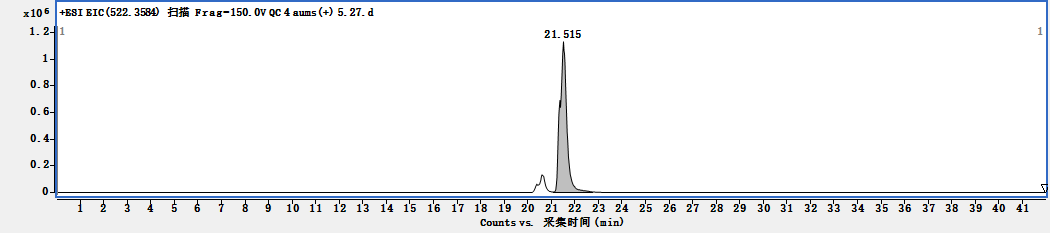

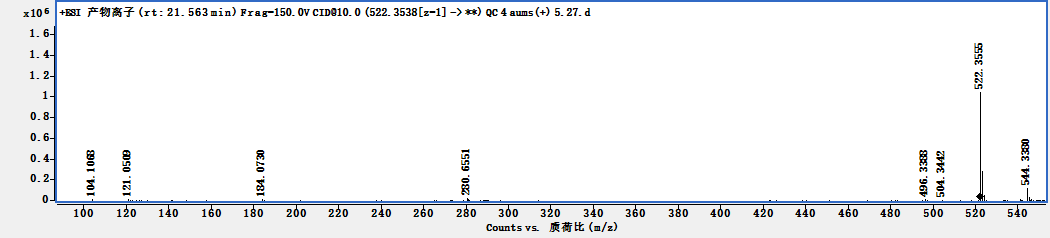

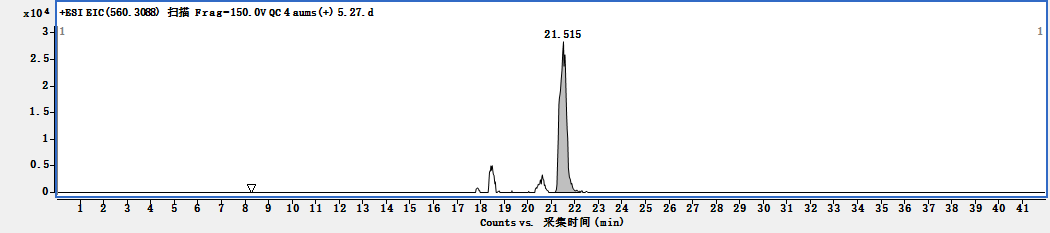

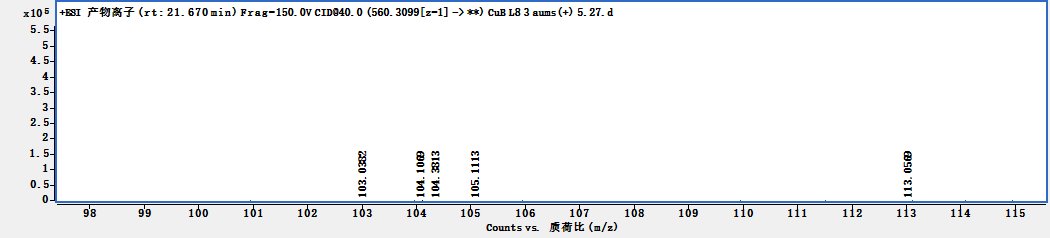

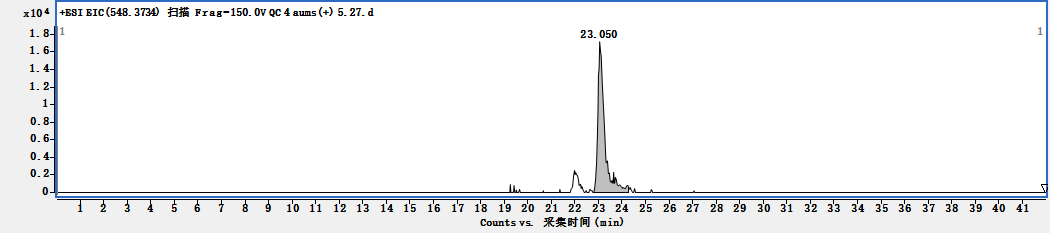

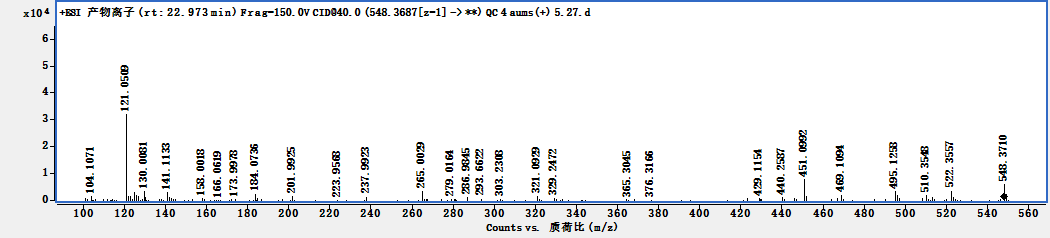

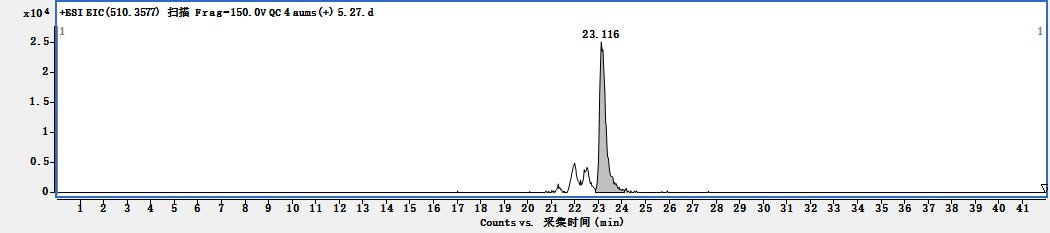

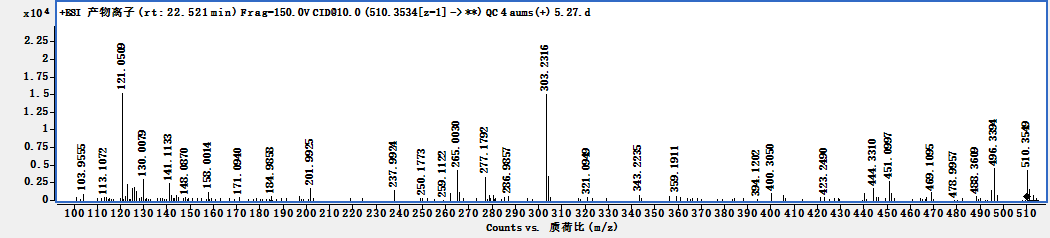

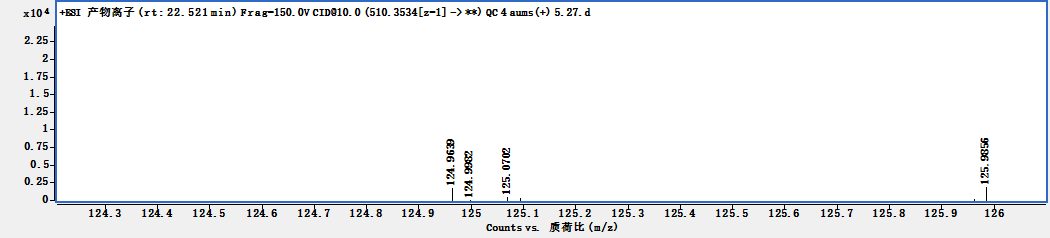

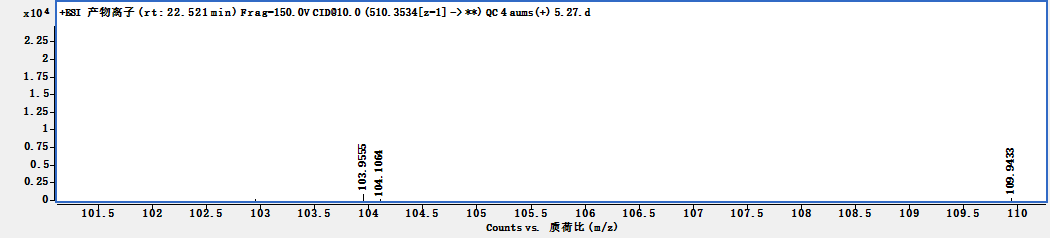

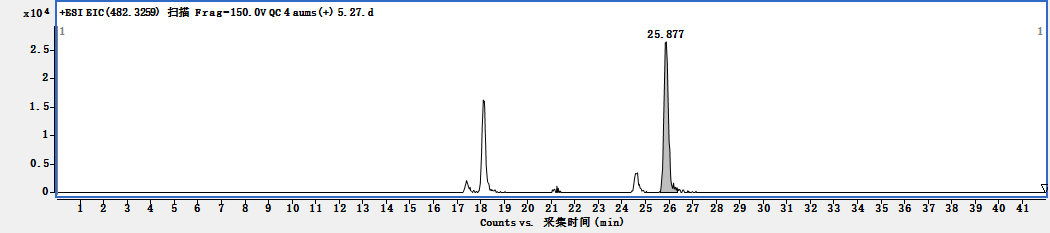

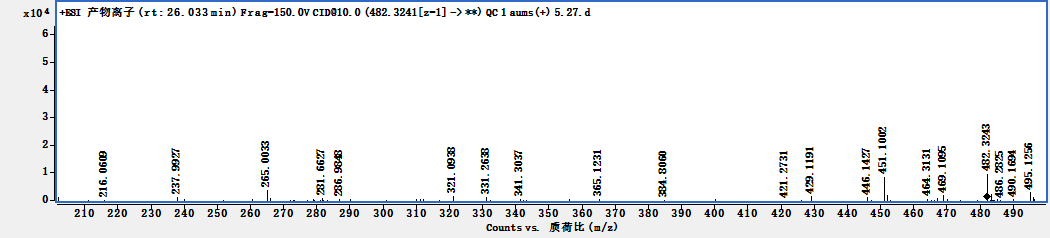

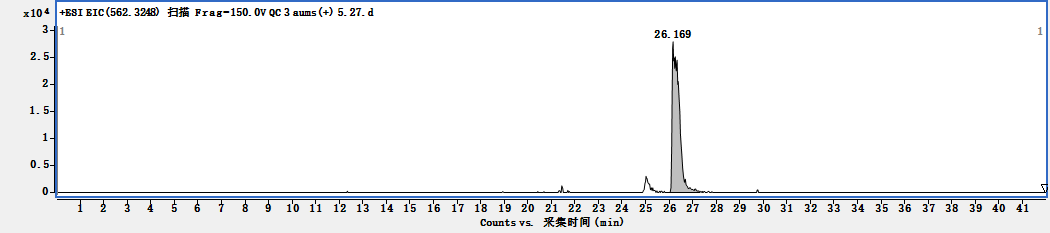

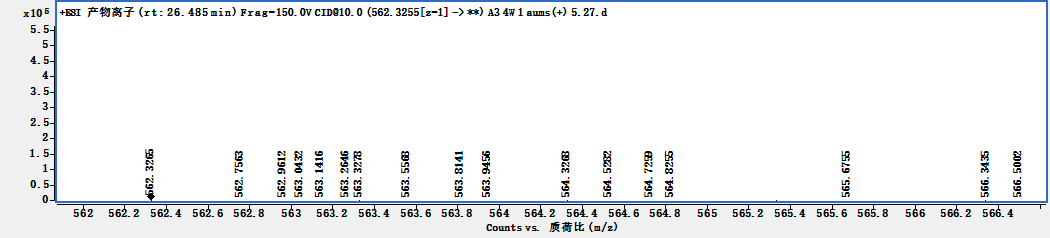

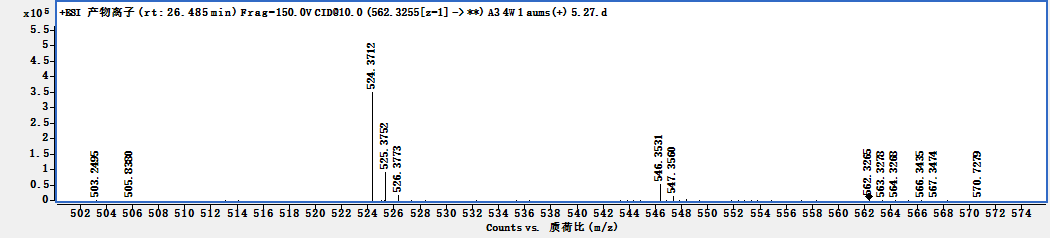

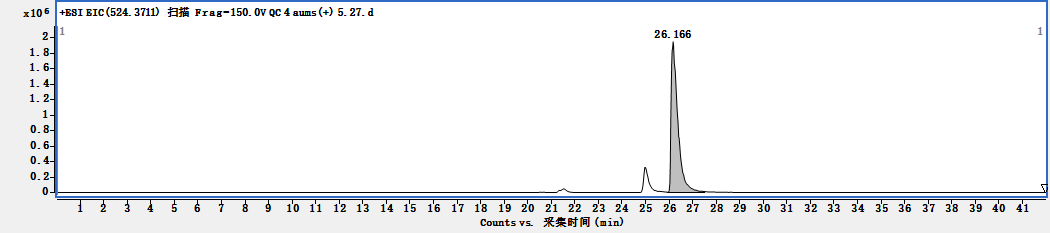

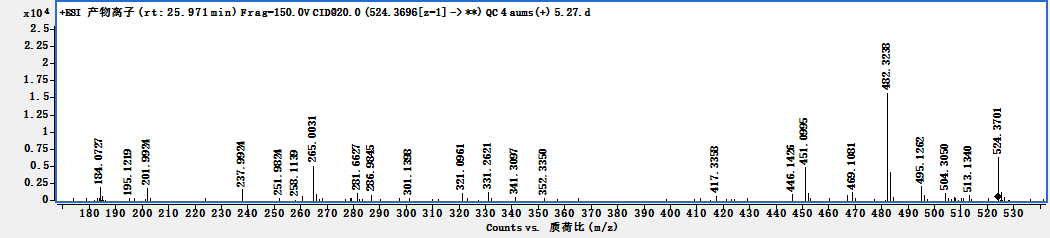

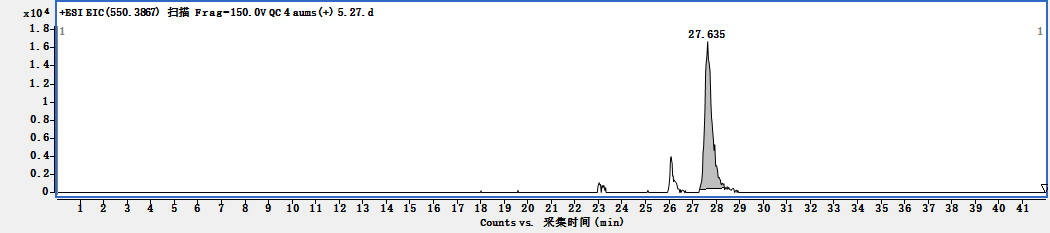

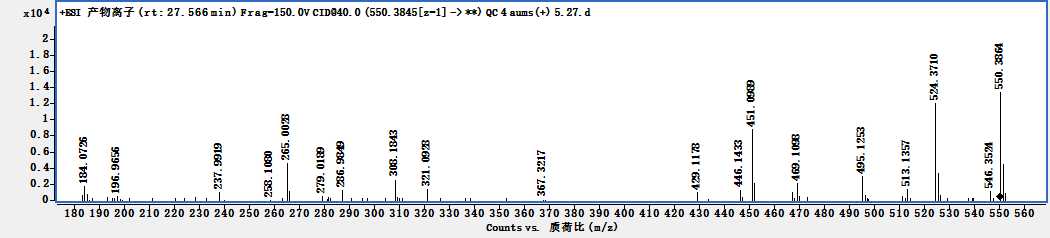


**6,8-Dihydroxypurine**

**Aminopropylcadaverine**

**Succinylacetoacetate**

**L-Norieucine**

**Hypoxanthine**

**Indoleacrylic acid**

**Deoxycytidine**

**N-Eicosapentaenoyl Glutamic acid**

**4-O-Methylmelleolide**

**Succinic acid**

**LysoPE(22:6(4Z,7Z,10Z,13Z,16Z,19Z)/0:0)**

**LysoPC(16:1(9Z)/0:0)**

**Guanosine diphosphate mannose**

**LysoPC(22:6(4Z,7Z,10Z,13Z,16Z,19Z)/0:0)**

**LysoPC(0:0/18:2(9Z,12Z))**

**LysoPC(20:4(8Z,11Z,14Z,17Z)/0:0)**

**LysoPE(20:4(8Z,11Z,14Z,17Z)/0:0)**

**Acrimarine N**

**LysoPC(20:5(5Z,8Z,11Z,14Z,17Z)/0:0)**

**LysoPE(16:0/0:0)**

**LysoPC(0:0/16:0)**

**LysoPE(22:2(13Z,16Z)/0:0)**

**LysoPC(22:5(7Z,10Z,13Z,16Z,19Z)/0:0)**

**LysoPC(20:3(8Z,11Z,14Z)/0:0)**

**Elaidic carnitine**

**Adenosine diphosphate ribose**

**LysoPC(18:1(9Z)/0:0)**

**LysoPC(17:0/0:0)**

**LysoPC(20:2(11Z,14Z)/0:0)**

**Protoporphyrin IX**

**LysoPE(0:0/18:0)**

**PC(18:1(9Z)e/2:0)**

**Platelet-activating factor**

**Negative mode**


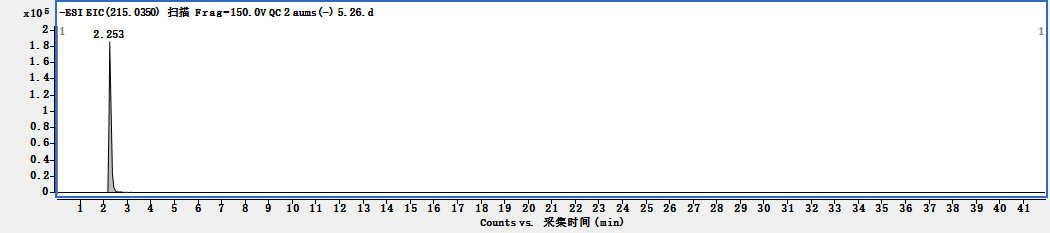

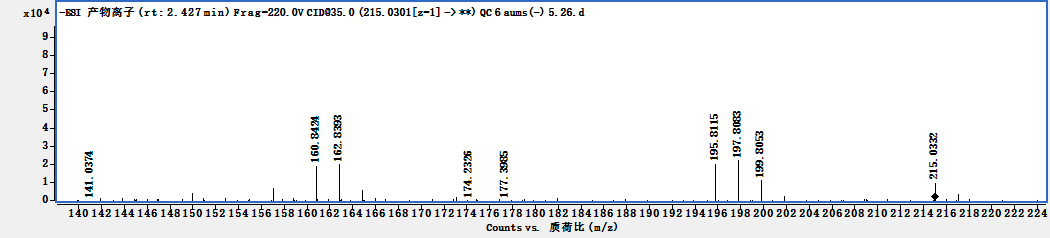

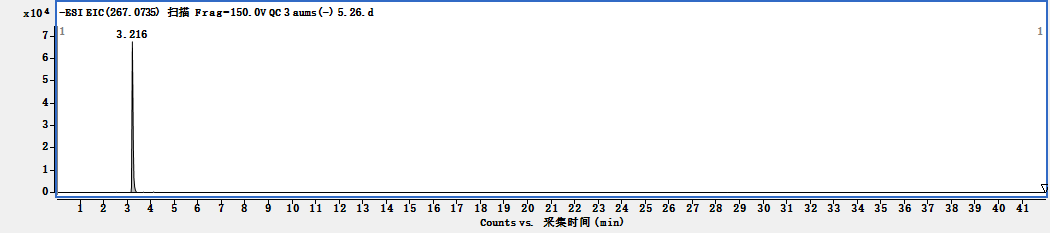

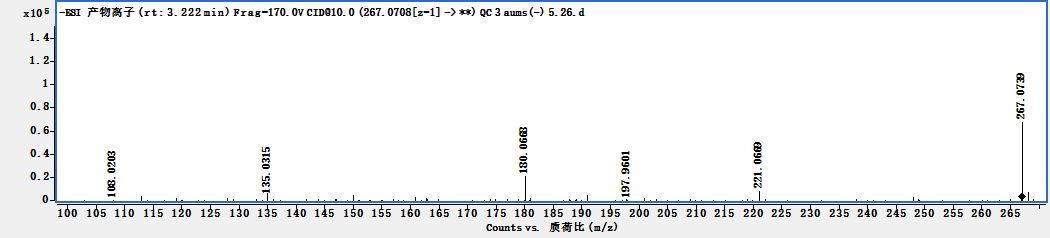

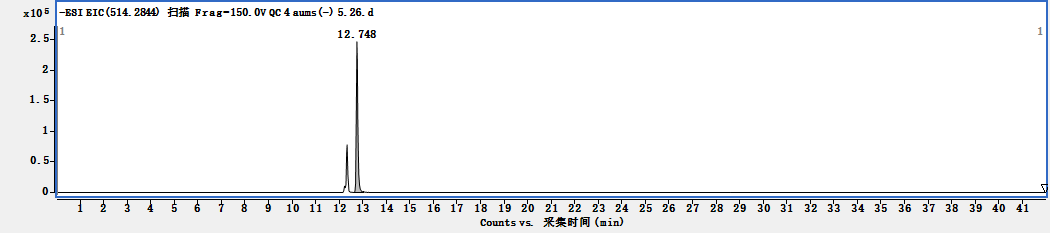

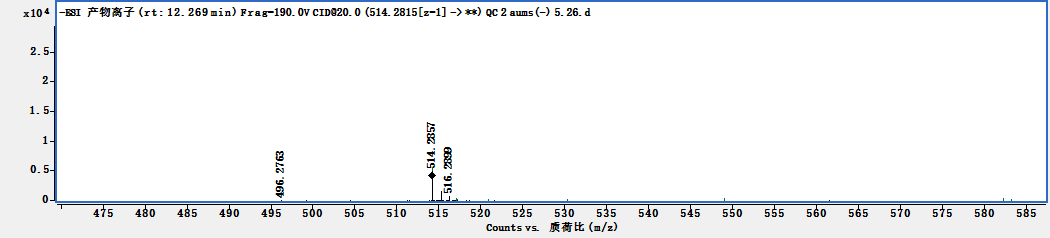

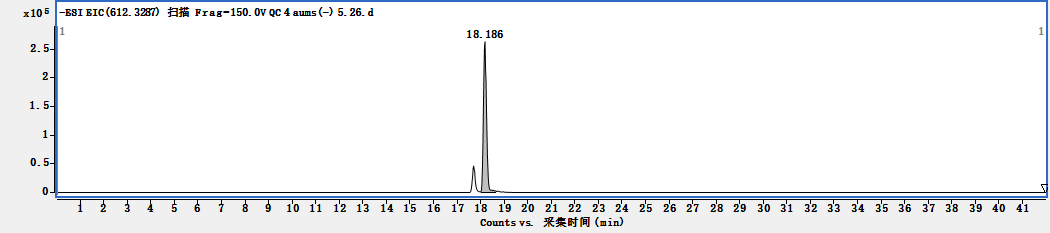

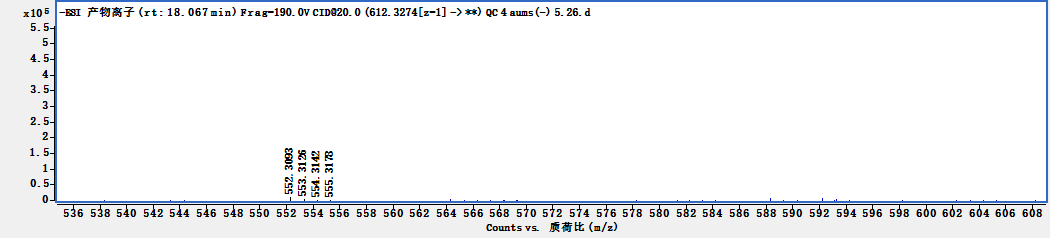

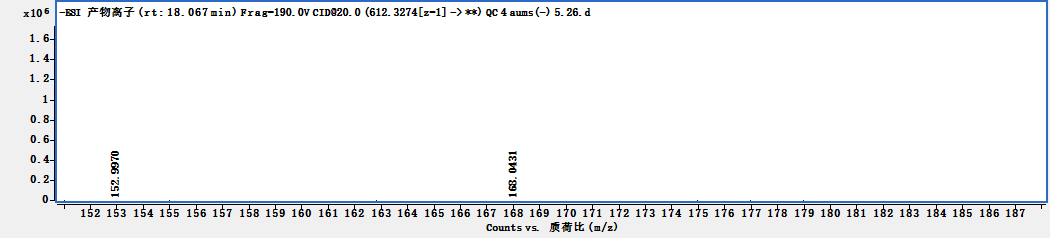

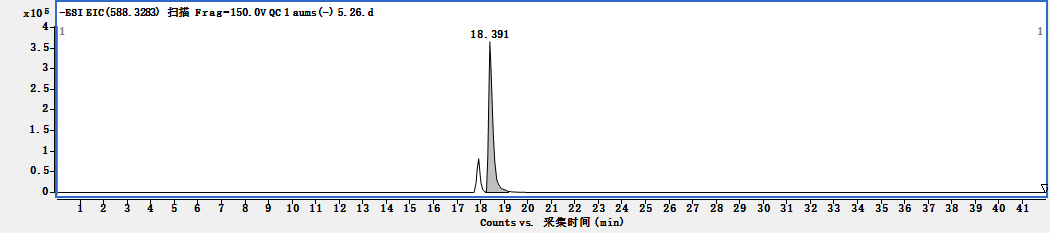

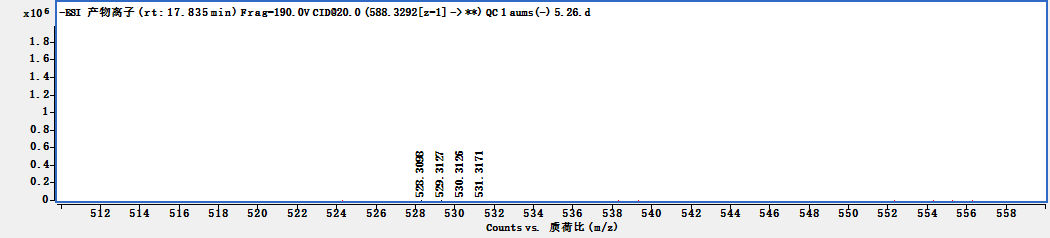

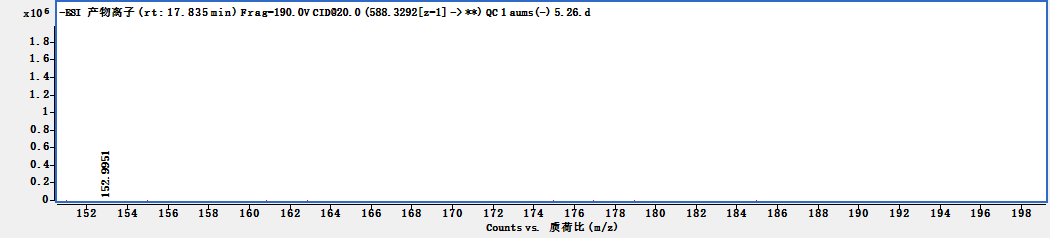

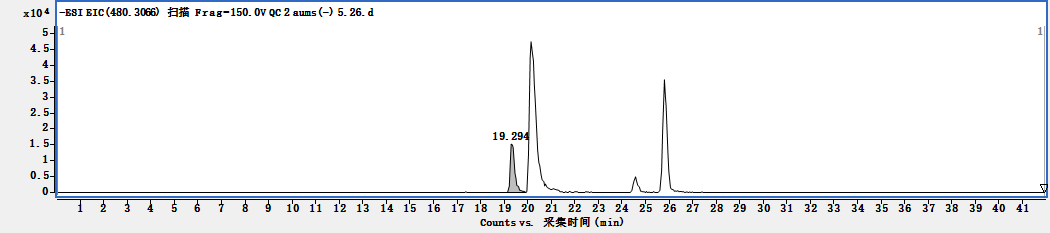

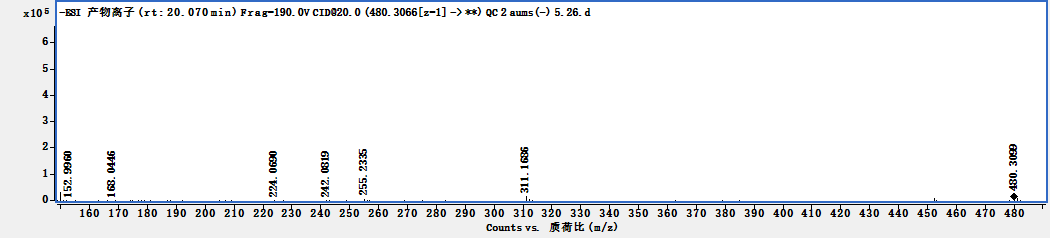

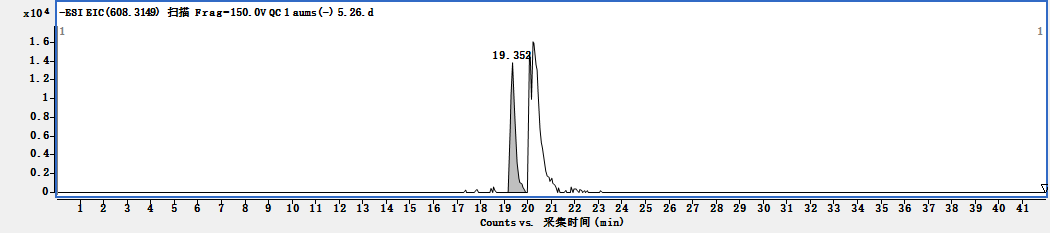

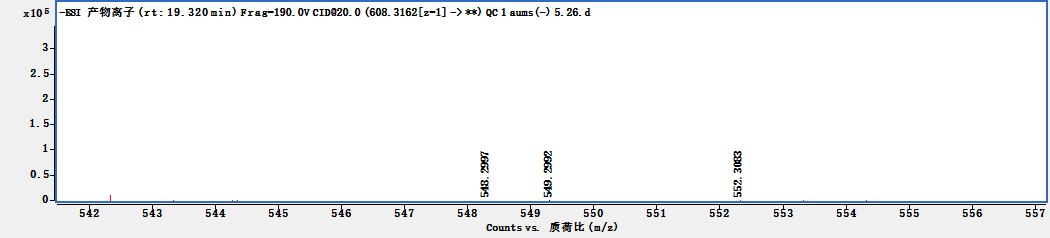

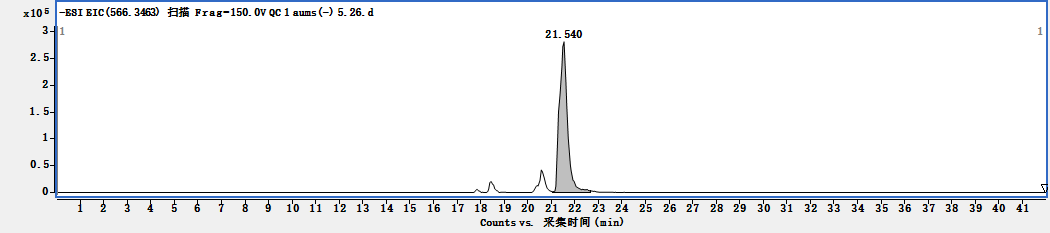


**Taurallocholic acid**

**Inosine**

**Bergapten**

**PE(22:4(7Z,10Z,13Z,16Z)/0:0)**

**LysoPE(24:6(6Z,9Z,12Z,15Z,18Z,21Z)/0:0)**

**PS(21:0/0:0)**

**PS(20:2(11Z,14Z)/0:0)**

**LysoPC(15:0/0:0)**

**PE(14:0/14:0)**

**Figure S6. The parent ions and corresponding fragment ion diagrams of several metabolites for HCC model in serum.**

**Pipecolic acid**

**Pipecolic acid**

**L-Methionine**

**3-Hydroxybutyrylcarnitine**

**Hypoxanthine**

**L-Methionine**

**Hypoxanthine**

**L-Tyrosine**

**Phenylpyruvic acid**

**S-Acetyldihydrolipoamide-E**

**Glutamylarginine**

**Sphingosine**

**LysoPE(18:2(9Z,12Z)/0:0)**

**N-Nervonoyl Serine**

**LysoPE(20:4(8Z,11Z,14Z,17Z)/0:0)**

**Pantetheine 4'-phosphate**

**Figure S7. The parent ions and corresponding fragment ion diagrams of several metabolites for HCC model in liver.**

**Figure S8. Enrichment diagram.** **A:** Control group VS early stage of HCC; **B:** Control group VS formative stage of HCC; **C:** formative stage of HCC VS CuB group.

**Figure S9. Heatmap visualization constructed based on the differential metabolites of importance of model. A, B:** serum samples; **C:** liver samples. The color blocks at different positions represent the relative expression levels of metabolites at the corresponding positions.

**Figure S10. TIC stacking diagram of CuB group of serum and liver in positive and negative ion mode. A, B:** serum samples; **C, D:** liver samples.

**Figure S11. Heatmap Analysis for CuB group. A:** serum samples; **B:** liver samples. The color blocks at different positions represent the relative expression levels of metabolites at the corresponding positions.
